# Stepwise GATA1 and SMC3 mutations alter megakaryocyte differentiation in a Down syndrome leukemia model

**DOI:** 10.1172/JCI156290

**Published:** 2022-07-15

**Authors:** Brahim Arkoun, Elie Robert, Fabien Boudia, Stefania Mazzi, Virginie Dufour, Aurélie Siret, Yasmine Mammasse, Zakia Aid, Matthieu Vieira, Aygun Imanci, Marine Aglave, Marie Cambot, Rachel Petermann, Sylvie Souquere, Philippe Rameau, Cyril Catelain, Romain Diot, Gérard Tachdjian, Olivier Hermine, Nathalie Droin, Najet Debili, Isabelle Plo, Sébastien Malinge, Eric Soler, Hana Raslova, Thomas Mercher, William Vainchenker

**Affiliations:** 1 INSERM, UMR1287, Gustave Roussy, Université Paris–Saclay, Equipe Labellisée Ligue Nationale Contre le Cancer, Villejuif, France.; 2 Laboratory of Excellence GReX, Université de Paris, Paris, France.; 3 INSERM, UMR1170, Gustave Roussy, Université Paris–Saclay, Equipe Labellisée Ligue Nationale Contre le Cancer, OPALE Carnot Institute, PEDIAC consortium, Villejuif, France.; 4 Institut National de la Transfusion Sanguine (INTS), Paris, France.; 5 Gustave Roussy, Plateforme de Bioinformatique,; 6 Gustave Roussy, Université Paris–Saclay, and; 7 Gustave Roussy, Plateforme Imagerie et Cytométrie, Université Paris–Saclay, UMS AMMICA, INSERM US23, CNRS UMS 3655, Villejuif, France.; 8 Assistance Publique-Hôpitaux de Paris, Service d’Histologie, Embryologie et Cytogénétique, Université Paris–Saclay, Hôpital Antoine Béclère, Clamart, France.; 9 INSERM U1163/CNRS ERL8254–Laboratory of Cellular and Molecular Mechanisms of Hematological Disorders and Therapeutic Implications, Institut Imagine, Paris, France.; 10 Gustave Roussy, Plateforme de Génomique, Université Paris–Saclay, UMS AMMICA, INSERM US23, CNRS UMS 3655, Villejuif, France.; 11 Telethon Kids Cancer Centre, Telethon Kids Institute, University of Western Australia, Perth, Western Australia, Australia.; 12 Institut de Génétique Moléculaire de Montpellier (IGMM), University of Montpellier, CNRS, Montpellier, France.

**Keywords:** Hematology, Oncology, Leukemias

## Abstract

Acute megakaryoblastic leukemia of Down syndrome (DS-AMKL) is a model of clonal evolution from a preleukemic transient myeloproliferative disorder requiring both a trisomy 21 (T21) and a *GATA1*s mutation to a leukemia driven by additional driver mutations. We modeled the megakaryocyte differentiation defect through stepwise gene editing of *GATA1s*, *SMC3^+/–^*, and *MPL^W515K^*, providing 20 different T21 or disomy 21 (D21) induced pluripotent stem cell (iPSC) clones. *GATA1s* profoundly reshaped iPSC-derived hematopoietic architecture with gradual myeloid-to-megakaryocyte shift and megakaryocyte differentiation alteration upon addition of *SMC3* and *MPL* mutations. Transcriptional, chromatin accessibility, and GATA1-binding data showed alteration of essential megakaryocyte differentiation genes, including *NFE2* downregulation that was associated with loss of GATA1s binding and functionally involved in megakaryocyte differentiation blockage. T21 enhanced the proliferative phenotype, reproducing the cellular and molecular abnormalities of DS-AMKL. Our study provides an array of human cell–based models revealing individual contributions of different mutations to DS-AMKL differentiation blockage, a major determinant of leukemic progression.

## Graphical abstract

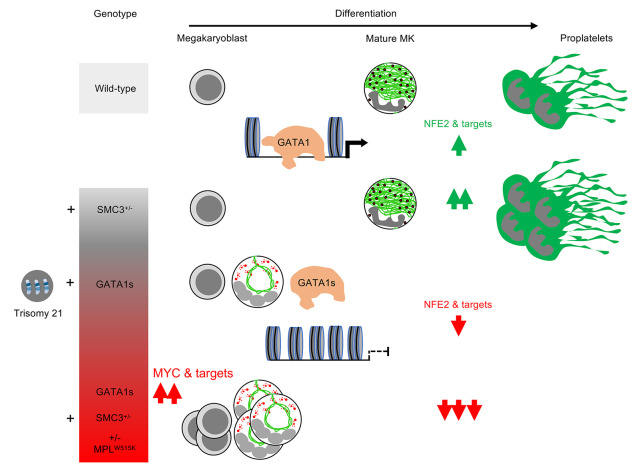

## Introduction

Acute megakaryoblastic leukemias (AMKLs) are rare disorders that are more frequent in children (1, 2). Children with Down syndrome (DS) who have constitutive trisomy 21 (T21) have an increased risk of developing AMKL (3). While children with DS-AMKL have unique phenotypic and clinical features, this condition also represents a model of multistep leukemogenesis with key characteristics found in many leukemias (4).

DS-AMKL is always preceded by a clonal preleukemic state called transient myeloproliferative disorder (TMD) driven by *GATA1* mutations, mostly in exon 2 and resulting in the expression of an N-terminal deleted shorter protein, called GATA1s (5, 6). Usually, TMD spontaneously regresses, but 10% to 20% of these children will develop DS-AMKL within 5 years (7). This leukemia originates from the initial *GATA1s* clone with 2 or more acquired additional mutations (8, 9), including loss-of-function mutations in cohesin complex genes (e.g., *STAG2*, *RAD21*, *SMC1A/3*) and a mutation in a gene encoding a signaling molecule leading to RAS or JAK/STAT pathway constitutive activation (8, 9). *EZH2* or *SUZ12*, 2 members of PRC2, are frequently mutated in addition to the other mutations.

How *GATA1s* and these secondary mutations cooperate to induce AMKL and why this leukemia development requires a T21 and a fetal hematopoiesis remain unclear and need modeling approaches. Mouse models have been limited by only minor defects induced by Gata1s in late megakaryocyte (MK) differentiation (10–13). Thus, it was crucial to develop human models of DS-AMKL. An approach consisting of introducing *GATA1s* in induced pluripotent stem cells (iPSCs) or deriving iPSCs from T21 individuals with and without TMD has been developed, but was mostly focused on the erythroid cells (14–17). A more recent approach has been to edit the *GATA1s* and *STAG2* mutations in primary human CD34^+^ cells, including T21 CD34^+^ fetal liver cells, followed by xenotransplantation (18, 19). The latter approach was focused on hematopoietic stem and progenitor cells (HSCP) and has clearly unveiled that the cooperation between *GATA1s* and *STAG2* mutations induces self-renewal and progression toward AMKL.

In contrast to erythroid differentiation (20, 21), the precise role of GATA1s on human MK differentiation is incompletely understood (17). It appears essential to better understanding how GATA1s precisely cooperates with T21 and additional mutations to induce MK differentiation blockage and proliferation, two key features of the leukemic process. Based on the consequences of cohesin mutations on HSPC (22–24), we hypothesized that *SMC3* haploinsufficiency may modify the chromatin occupancy of GATA1s and other transcription factors regulating late MK differentiation. We modeled the effects of different mutations implicated in the progression of TMD to AMKL on human hematopoiesis and more particularly on the MK differentiation. For this purpose, we sequentially introduced *GATA1s*, a gain-of-function *MPL* mutation (*MPL^W515K^*) (25, 26) and a heterozygous inactivation of *SMC3* (*SMC3^+/–^*), using CRISPR-Cas9 technology in T21 iPSC. Moreover, to understand the role of T21, we contrasted disomic 21 (D21) with T21 iPSCs. This approach provides insights at the cellular and molecular levels on how GATA1s alters MK differentiation alone or in cooperation with SMC3 deficiency to deeply enhance differentiation alterations and increase proliferation.

## Results

### Sequential editing generates a panel of iPSC lines to investigate the different steps of DS-AMKL progression.

**Figure 1:**
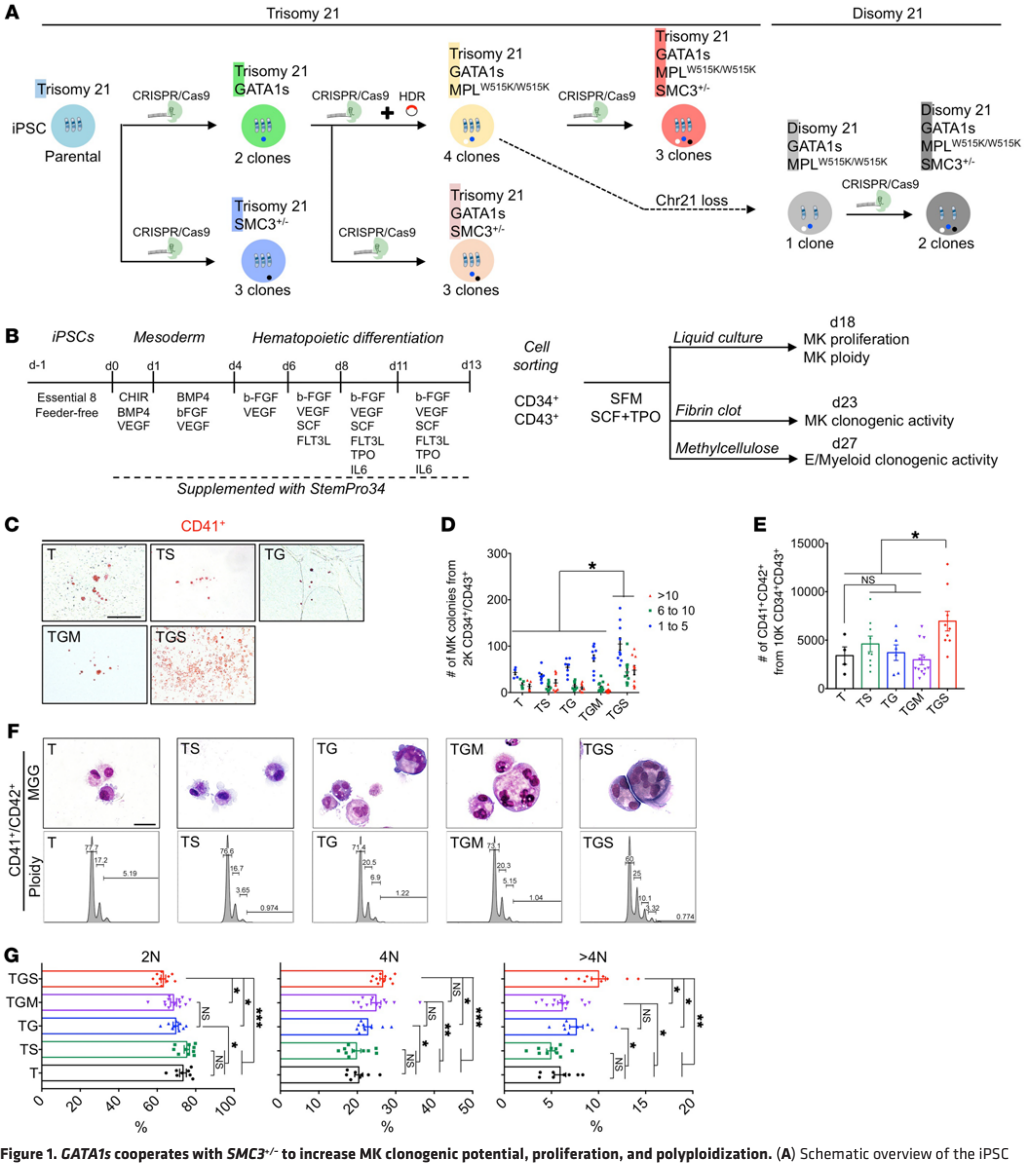
*GATA1s* cooperates with *SMC3^+/–^* to increase MK clonogenic potential, proliferation, and polyploidization. (**A**) Schematic overview of the iPSC clones generated by a stepwise introduction of *GATA1s*, *MPL^W515K^*, and *SMC3^+/–^*, using CRISPR/Cas9. Bold letters highlight the abbreviation used hereafter. The number of clones obtained for each genotype and subjected to MK differentiation studies is indicated. The dashed arrow indicates the isogenic D iPSC clone harboring *GATA1s* and *MPL^W515K^* mutations randomly obtained through loss of 1 chromosome 21. (**B**) Schematic overview of the hematopoietic differentiation method used and the subsequent MK phenotypic characterization. (**C**) Representative images of CFU-MK colonies. Scale bar: 500 μm. (**D**) Histogram of the number of CD41^+^ colonies obtained from 2000 CD34^+^CD43^+^ in fibrin clot assay. (**E**) Histogram of the number of CD41^+^CD42^+^ MKs obtained from 10,000 CD34^+^CD43^+^ in liquid culture assay. Data are represented as mean ± SEM; *n* = 3–4. The number of clones tested per genotype was as follows: T/parental = 1; TS = 3; TG = 2; TGM = 4; TGS = 3. (**F**) May-Grünwald-Giemsa (MGG) staining (**E**, upper panels) and ploidy plots (**E**, lower panels) of iMK according to the indicated genotypes. T and TS CD41^+^CD42^+^ showed typically mature micro-MKs with acidophilic cytoplasm, while TG, TGM, or TGS showed large polyploid immature MKs with basophilic cytoplasm. Scale bar: 50 μm. (**G**) Histograms of the percentages of 2N, 4N, and >4N of iMKs. Data are represented as mean ± SEM; *n* = 3 to 8. The number of clones tested per genotype was as follows: T/parental = 1; TS = 2; TG = 2; TGM = 4; TGS = 3. Statistical significance was determined using 1-tailed Mann-Whitney *U* test. *P < 0.05; **P < 0.01; ***P < 0.001.

To recapitulate DS-AMKL progression, we sequentially introduced by CRISPR/Cas9-mediated engineering *GATA1s* mutations (G), followed by an *MPL^W515K^* mutation (M) and *SMC3* mutations (S), into the parental T iPSCs (Figure 1A).

First, we generated 2 Trisomy 21 GATA1s (TG) iPSC clones carrying *GATA1s* truncating mutations and confirmed that T MKs expressed both full-length (FL) and short GATA1 isoforms, while TG mutant MKs expressed only GATA1s (Supplemental Figure 1, A, B, and R; supplemental material available online with this article; https://doi.org/10.1172/JCI156290DS1). Thereafter, the *MPL^W515K^* gain-of-function mutation was introduced in a TG clone to generate 4 Trisomy 21 GATA1s *MPL^W515K/W515K^* (TGM) clones (Supplemental Figure 1, C–F and S). To assess the functionality of the *MPL^W515K^* mutation, we quantified the MK differentiation (% of CD41^+^CD42^+^ cells) from *MPL^WT/WT^* and *MPL^W515K/W515K^* iPSCs (Supplemental Figure 1, G and H). The MK differentiation obtained from the TGM in the presence of Stem Cell Factor (SCF) was Thrombopoietin (TPO) independent and abolished by ruxolitinib, a JAK1/JAK2 inhibitor, demonstrating that the MPL*^W515K^* mutant was functional.

To assess the role of *SMC3* haploinsufficiency, we edited *SMC3* in the heterozygous state in T, TG, and TGM and we obtained 3 clones in each genetic context (TS, TGS, TGMS) (Supplemental Figure 1, I–K). *SMC3* haploinsufficiency was confirmed by Western blots (Supplemental Figure 1, L and M). Notably, intracellular flow cytometry analysis indicated a similar increase in GATA1 protein levels in MKs from TG and TGMS as compared with T (Supplemental Figure 1, N and O).

To understand the role of T21, we derived an isogenic D *GATA1s* *MPL^W515K^* (DGM) clone by a spontaneous loss of a chromosome 21 (Supplemental Figure 1V). Then we generated 2 isogenic DGMS clones (Supplemental Figure 1, T and V). MKs from DGMS and TGMS presented similar levels of GATA1 protein (Supplemental Figure 1, P and Q).

All iPSC clones were genetically characterized by karyotypic analyses (Supplemental Figure 1, U and V), fluorescence in situ hybridization (Supplemental Figure 1W), and Sanger sequencing (no off-targets in CRISPR/Cas9-edited iPSC clones; Supplemental Table 1). We obtained a panel of 20 different iPSC clones, presenting functionally validated mutations in *GATA1*, *MPL*, and *SMC3* that allowed us to study the contribution of each mutation, including T21, and those mutations’ cooperation in the progression to DS-AMKL.

### GATA1s and SMC3^+/–^ alter hematopoietic progenitors and MK yield.

To investigate the hematopoietic differentiation of the different iPSC lines, we used a 2D cell culture with a sequential addition of cytokines (Figure 1B) and CD34^+^CD43^+^ hematopoietic progenitor cells (HPC) purified from the supernatant at day 13 of culture. While T, TS clones generated erythroid and myeloid colonies, TG clones showed increased myeloid, but abrogated erythroid, colony formation (Supplemental Figure 2, A and B), confirming previous data (16). The addition of *MPL^W515K/W515K^* and/or *SMC3^+/–^* had no significant effect (Supplemental Figure 2, A and B).

The MK colony-forming potential, the size of MK colonies, and the total number of generated CD41^+^CD42^+^ MKs were significantly increased in T21 only when *GATA1s* and *SMC3^+/–^* were combined (Figure 1, C–E). T and TS showed typical micro-MK (Figure 1F), characteristic for the iPSC-derived MKs (iMKs). However, all genotypes presenting GATA1s showed the presence of large-sized basophilic dysplastic multilobulated MKs (Figure 1F). The predominant classes of ploidy were the 2N and 4N, as observed for MK derived from WT human iPSCs or embryonic stem (ES) cells (27). GATA1s increased the mean percentage of more than 4N MKs in synergy with S, but not M (Figure 1, F and G). These data support the idea that GATA1s cooperates with *SMC3* haploinsufficiency in T21 to alter MK differentiation, including increased clonogenic potential and proliferation of MK progenitors with accumulation at an immature stage and increased size and ploidy in some immature MKs.

### Effects of SMC3^+/–^ and GATA1s on MK maturation.

**Figure 2:**
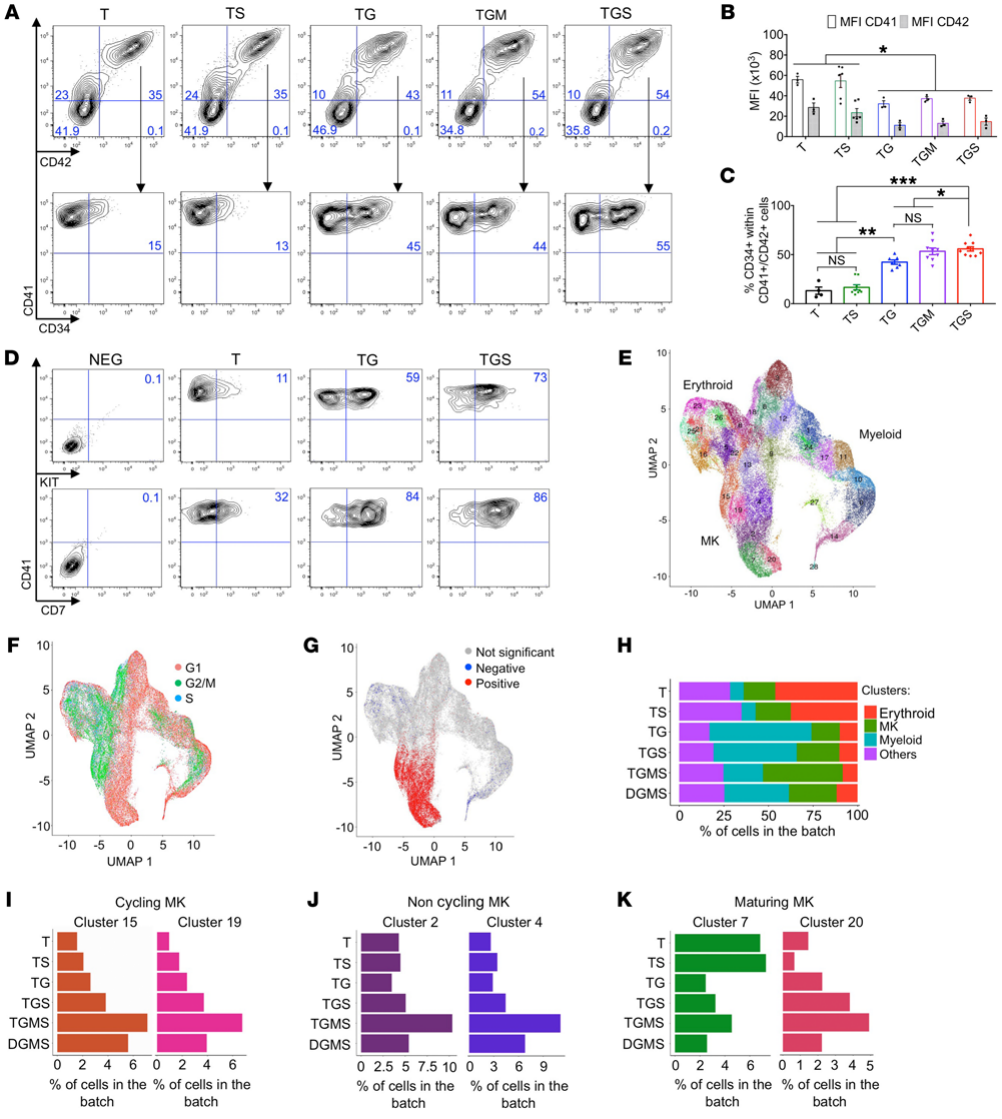
Assessment of iMK differentiation alterations. (**A**–**D**) Immunophenotypes of iMKs for the CD34, KIT, CD7, CD41, and CD42 markers found in DS-AMKL patient blasts. (**A**) Contour plots showing the expression of CD34, CD41, and CD42 markers. (**B**) Histogram shows the MFI of CD41 and CD42. (**C**) Histogram shows the percentage of CD34^+^CD41^+^ per total CD41^+^ population. Data in **B** and **C** are represented as mean ± SEM; *n* = 3–4. The number of clones tested per genotype was as follows: T/parental = 1; TS = 2; TG = 2; TGM = 3; TGS = 3. Statistical significance was determined using 1-tailed Mann-Whitney’s *U* test: *P < 0.05; **P < 0.01; ***P < 0.001. (**D**) Representative contour plots of KIT and CD7 expression in the iMK population from 2 independent experiments. (**E** and **F**) scRNA-Seq of CD43^+^ iPSC-derived hematopoietic cells at day 13 of culture. (**E**) UMAP integration of cells from all conditions. Clusters were defined using the Louvain algorithm and numbered and labeled with unique colors. (**F**) UMAP integration with cells colored according to the predicted cell-cycle stage (Seurat method). (**G**) UMAP integration with cells colored according to the enrichment in a MK signature. Red, cells are significantly enriched for the signature; blue, cells are significantly depleted for the signature; gray, no significant enrichment. (**H**) Bar plot shows the proportion of cells in the indicated hematopoietic lineages for each condition. (**I**) Bar plots of the proportion of cells in the 2 clusters of cycling MKs. (**J**) Bar plots of the proportion of cells in the 2 clusters of noncycling MKs. (**K**) Bar plots of the proportion of cells in the 2 clusters of maturing MKs. Cluster 7 represents normal maturing MKs. Cluster 20 represents an abnormal MK population.

We then assessed cell-surface marker expression. While a fraction of CD41^+^ cells were CD42 negative, as revealed by an anti-CD42a antibody, the vast majority expressed the CD42 from all genotypes (Figure 2, A and B), including an independent D21 iPSC derived from a healthy individual (Supplemental Figure 3, A and B). This phenotype matches what is observed in the MK differentiation of iPSCs or primary CD34^+^ cells. However, MFI was decreased by *GATA1s* independently of the associated mutations, suggesting that GATA1s impaired terminal MK maturation (Figure 2, A and B). The CD41^+^CD42^+^ cell fractions that maintained CD34 expression were 30% and 10% in D21 and T21, respectively (Supplemental Figure 3C). CD34 expression was increased by GATA1s in T21 (TG) to 45% and further by *SMC3* haploinsufficiency to 55% (Figure 2, A and C). TMD and DS-AMKL megakaryoblasts are characterized by the expression of CD34, CD41, and CD42 antigens, but also other markers, such as KIT and CD7 (28, 29). While only a small fraction of D21 or T21 CD41^+^ cells expresses KIT (Supplemental Figure 3A), it is detected on over half of the *GATA1s* CD41^+^ cells and further increased by *SMC3* haploinsufficiency (Figure 2D). Similar results were observed for CD7 (Supplemental Figure 3A and Figure 2D). These results show that the GATA1s-induced blockage of MK differentiation at the megakaryoblast stage is worsened by *SMC3* haploinsufficiency.

To obtain a precise picture of the altered differentiation, we performed single-cell RNA-Seq (scRNA-Seq) for D, T, TS, TG, TGS, TGMS, and DGMS at an early differentiation time point (day 13). From 6800 to 14,001 cells with similar characteristics were analyzed per condition (Supplemental Figure 4A). UMAP clustering (https://umap-learn.readthedocs.io/en/latest/clustering.html) obtained by pooling cells from all the conditions provided 28 clusters (Figure 2E) for which cell-cycle status was inferred using Seurat (Figure 2F and Supplemental Figure 5A) and cells of the different clusters showed expression of MK, erythroid, and myeloid differentiation markers (Figure 2G, Supplemental Figure 4, B–D, and Supplemental Figure 5B). To identify all cells belonging to the MK, erythroid, and myeloid lineages, we computed scores for lineage-specific gene lists (Supplemental Figure 4E). T21 differentiation was clearly biased toward the erythroid lineage in comparison with D21 (Supplemental Figure 5C). The highest percentage of MK cells was observed in TGMS (Figure 2H). By quantifying the clusters along MK differentiation, we observed that the cycling, noncycling, and maturing MKs were similar between D21 and T21 (Supplemental Figure 5, D–F). Interestingly, a gradual increase for the cycling MKs (clusters 15 and 19) was observed in TS, TG, TGS, and TGMS as compared with T, with the highest percentage in TGMS (Figure 2I). Noncycling MK clusters (clusters 2 and 4) were more specifically amplified in TGMS as compared with T, TS, TG, and TGS (Figure 2J). Strikingly, 2 clusters were associated with the highest expression of maturing MK markers (clusters 7 and 20) (Figure 2K). Cluster 7 represented the vast majority of cells in T and TS, while cluster 20 was gradually increased in TG, TGS, and TGMS, representing approximately 50% of maturing MKs in TGMS.

Together, these data show that GATA1s and *SMC3^+/–^* cooperated together and with T21 through (a) an increase in immature cycling MKs and (b) an altered MK differentiation with the predominance of a cluster of maturing MKs that represents a minority among D21 or T21 MKs. MPL*^W515K^* markedly increased MK output at the expense of the myeloid lineage.

### GATA1s impairs organelle development and proplatelet formation.

**Figure 3:**
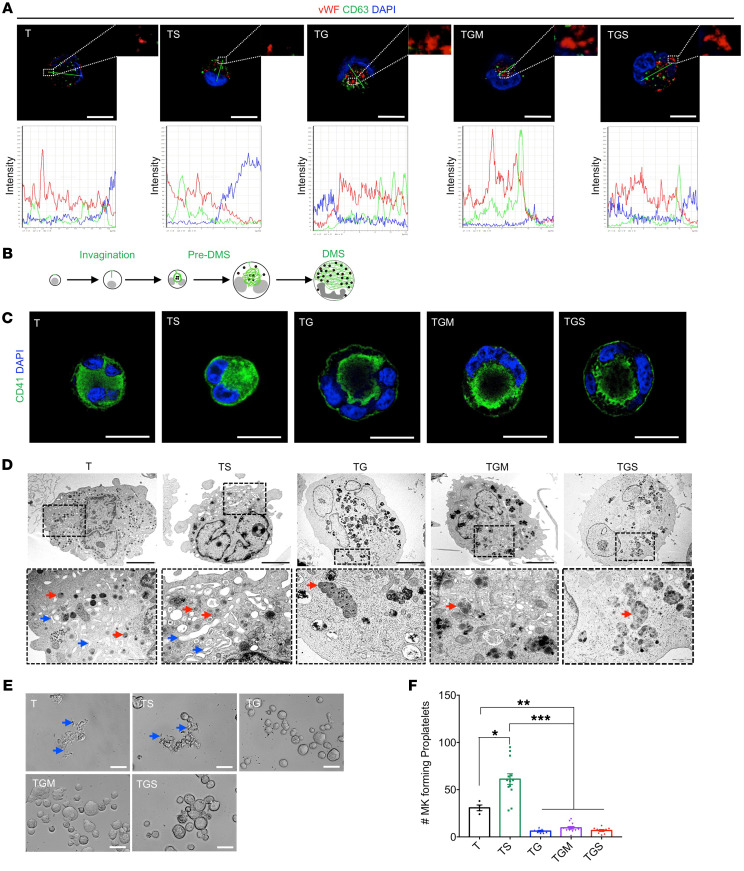
Assessment of MK maturation and platelet formation. (**A**) Confocal analyses of vWF and CD63 expression in MK (left panel) and colocalization analyses (right panel). Scale bars: 50 μm. (**B**) Schematic overview of the normal development steps of DMS. (**C**) Confocal analysis of CD41 marker distribution in iMKs. Scale bars: 50 μm. (**D**) Ultrastructural characterization of the iMK according to the compared genotypes. A representative MK is shown for each condition (upper panels), a part of which (dotted square) is enlarged (lower panels). More than 80% of T and TS MKs showed maturity characteristics associated with condensed nuclei, a well-developed DMS (blue arrows), and the presence of normal α-granules (red arrows). More than 90% of TG, TGM, and TGS MKs showed blockages in maturation characterized by the presence of uncondensed nuclei majorly composed of euchromatin and the absence of DMS formation, with endosomes presenting an abnormal accumulation of granules (red arrows). Scale bars: 5 μm. (**E**) Representative microphotographs of CD41^+^CD42^+^ iMKs under PPT formation assay. PPT-forming MKs are highlighted with blue arrows in the T and TS conditions. Scale bars: 50 μm. (**F**) Histogram of the number of PPT-forming MKs in the compared conditions. Data are represented as mean ± SEM; *n* = 3–4. The number of clones tested per genotype was as follows: T/parental = 1; TS = 3; TG = 2; TGM = 4; TGS = 3. Statistical significance was determined using 1-tailed Mann-Whitney’s *U* test. *P < 0.05; **P < 0.01; ***P < 0.001.

To better understand the cellular defect in MK differentiation, we performed staining of vWF and CD63, respective markers of α-granules and lysosomes, both deriving from multivesicular bodies (Figure 3A). In contrast with T and TS MKs, TG MKs showed the presence of abnormally large sized α-granules, as attested by the vWF labeling (Figure 3A). Colabeling with the anti-CD63 antibody was as rare as in control MKs (10% of total MK), attesting that these large granules were not multivesicular bodies (Figure 3A). The anti-CD41 antibody was used to highlight demarcation membrane system (DMS) development (Figure 3B; adapted from ref. 30). In T and TS MKs, CD41 was localized at the cell surface and well distributed in the cytoplasm (Figure 3C) in more than 90% of the MKs. In contrast, in the presence of *GATA1s*, CD41 was detected in the center of the cell between the nuclear lobes and only very weakly at the cell surface in the great majority of the cells (Figure 3C). Complete *Z*-stack analysis showed that this intracytoplasmic labeling was in continuity with the cell surface with usually only a single connection demonstrating the presence of a rudimental DMS. This abnormality was even increased by *SMC3^+/–^* with a more polarized labeling (Figure 3C). Ultrastructural studies confirmed that the *GATA1s* mutation was associated with the presence of very large cells (over 40 μm diameter) with a large multilobulated uncondensed nucleus containing a majority of euchromatin and large atypical granules (Figure 3D). Only a pre-DMS could be detected, usually localized between the nuclear lobes, a defect that was increased by *SMC3*^+/–^.

Then, we investigated the effects of GATA1s and the other mutations on proplatelet (PPT) formation. T and TS MKs were able to form PPTs (Figure 3E). Unexpectedly, *SMC3^+/–^* increased the percentage of PPT-forming MKs, suggesting that, in the context of GATA1^WT^, it favors MK maturation (Figure 3F). In contrast, MKs from all *GATA1s* conditions, including with *SMC3^+/–^*, barely formed PPT (Figure 3, E and F).

### Combined GATA1s and SMC3^+/–^ induce proliferative and DS-AMKL signatures.

We then performed transcriptomic analyses. To study a pure population of MKs, we excluded cells positive for the CD33 myeloid marker and sorted CD41^+^CD42^+^CD33^–^ cells using 2 consecutive sorting steps to achieve a purity greater than 98%, confirmed by flow cytometry reanalysis and cytology (Supplemental Figure 6, A–C). By principal component analysis (PCA), a clear clustering of samples according to GATA1s or *SMC3^+/–^* status was found (Supplemental Figure 6D) with a large number of deregulated genes (DEGs) in TG versus T and TS, respectively (adjusted *P* < 0.05; Supplemental Figure 6E). MPL*^W515K^* did not influence the clustering and had little impact on gene expression (Supplemental Figure 6, D and E). In contrast, TGS importantly increased the number of DEGs when compared with T, TS, and TG, respectively (Supplemental Figure 6E).

**Figure 4:**
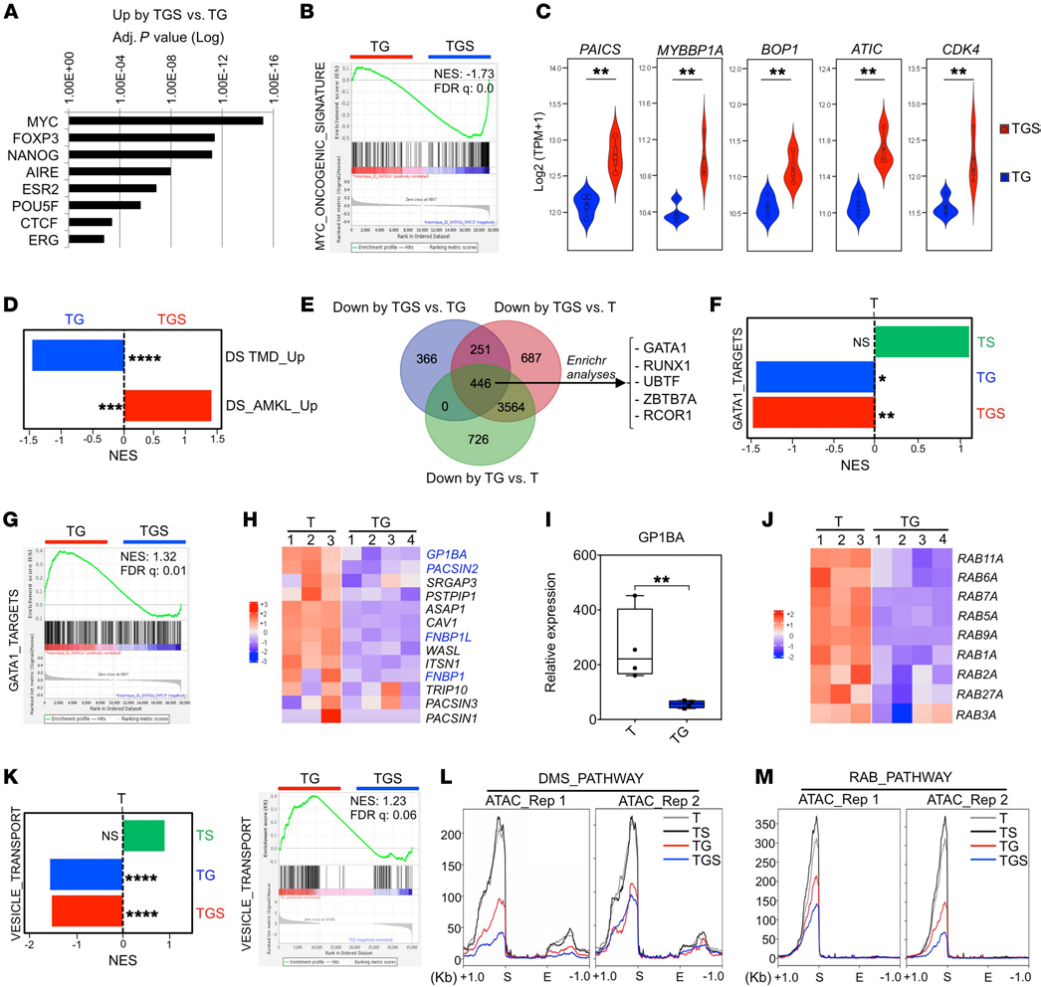
*GATA1s* cooperates with *SMC3^+/–^* to acquire DS-AMKL features in iMK. (**A**) Transcriptional signature (transcription factor protein-protein interactions) of the upregulated genes in TGS versus TG iMKs. (**B**) GSEA of the MYC oncogenic signature in TGS versus TG. (**C**) Violin plots showing expression (GSEA) of the MYC target genes in TGS versus TG. **P < 0.01. (**D**) GSEA of the genes upregulated in DS-TMD compared with DS-AMKL or the genes upregulated in DS-AMKL compared with DS-TMD (31) patients in TGS versus TG iMKs. ***FDR < 0.01; ****FDR < 0.001. (**E**) Venn diagram showing the overlap of downregulated genes in the indicated comparisons. The top transcriptional signatures from the commonly downregulated genes in the compared conditions are annotated with an adjusted *P* < 0.000001 (Encode and ChEA Consensus TFs from CHIP-X). (**F**) GSEA for *GATA1* gene targets (32) in TS versus T, TG versus T, and TGS versus T iMK. NES, normalized enrichment score. *FDR < 0.25; **FDR < 0.05. (**G**) GSEA for *GATA1* gene targets (32) in TGS versus TG iMKs. (**H**) Heatmap showing the expression of genes encoding the F-BAR domain-containing proteins in T versus TG iMKs. Genes known to be direct targets of GATA1 (32) are marked in blue. (**I**) RT-qPCR analysis of *GP1BA* relative expression in T versus TG iMKs. **P < 0.01. (**J**) Heatmap showing the expression of the RAB family genes in T versus TG iMKs. (**K**) GSEA for vesicle-mediated transport (GO term GO:0016192) in T versus TG or TG versus TGS iMKs. ****FDR < 0.001. (**L**) Profile plots of ATAC signal on DMS genes comparing T, TS, TG, and TGS iMKs. The start (S) and the end (E) of the genes were plotted across a 1 kb flanking window; the *y* axis indicates depth per million mapped reads. Left panel: replicate (Rep) 1; right panel: replicate 2. (**M**) Profile plots of ATAC signal on RAB family genes comparing T, TS, TG, and TGS iMKs.

The genes commonly upregulated by TGS versus T, TS, or TG were enriched in signatures associated with cell-cycle activity and DNA replication (Supplemental Figure 6F), including MYC signatures (Figure 4, A and B) and MYC target genes (e.g. *PAICS*, *MYBP1A*, *BOP1*, *ATIC*, and *CDK4*) (Figure 4C). Importantly, Gene Set Enrichment Analyses (GSEAs) indicated that the gene signature characterizing DS-AMKL versus DS-TMD blast patients (31) was also enriched in the TGS versus TG MKs (Figure 4D). Accordingly, the gene signature of DS-TMD versus DS-AMKL patients was enriched in TG versus TGS MKs (Figure 4D). These results support the idea that *SMC3* haploinsufficiency acquisition, in the context of T21 and GATA1s, leads to increased proliferation properties and reproduces the molecular progression observed from TMD to DS-AMKL in patients.

### SMC3^+/–^ cooperates with GATA1s to impair GATA1 target and MK maturation gene expression.

We subsequently analyzed GATA1 target genes involved in MK maturation. Gene Ontology and GSEA analyses indicated that GATA1 target genes (32) are downregulated in TG versus T (Figure 4, E and F). *SMC3^+/–^* further decreased expression of GATA targets when associated with GATA1s (Figure 4, E and G), but had no significant effect on GATA1 targets in a GATA1^WT^ context (Figure 4F). Genes involved in DMS development, such as *GP1BA* and *PACSIN2* (Figure 4, H and I), and genes encoding the RAB family proteins controlling membrane trafficking and granule movement were downregulated in TG versus T MKs (Figure 4J). In addition, TG MKs were depleted in genes involved in vesicle transport compared with T MKs, and addition of *SMC3^+/–^* further depleted this signature (Figure 4K).

In view of these marked differences between GATA1 and GATA1s, we hypothesized that GATA1 and GATA1s may modify chromatin accessibility at key differentiation gene loci. Addition of *GATA1s* (TG), but not *SMC3^+/–^* (TS), significantly reduced the global intensity of ATAC-Seq signals compared with T (Supplemental Figure 7A). Combination of *GATA1s* and *SMC3^+/–^* showed further reduction in peak intensity (Supplemental Figure 7A). Lower accessibilities in TG versus T and in TGS versus T were seen in promoter, intergenic, and intronic regions, while the gained sites were mainly intergenic and intronic (Supplemental Figure 7B). The peak distribution was different in GATA1^WT^ (TS versus T) (Supplemental Figure 7B). ATAC profiles on DMS and RAB pathway genes showed a decreased accessibility at their transcription starting sites in TG and even more in TGS versus T (Figure 4, L and M). Together, combined RNA-Seq and ATAC-Seq profiles show that GATA1s in T MKs represses GATA1 targets as well as genes involved in organelle formation, a phenotype that was further enhanced by addition of *SMC3^+/–^*, supporting a model of cooperation leading to a failure of the GATA1-driven transcriptional program.

### Molecular signatures of progression identify inhibition of the NFE2 program.

**Figure 5:**
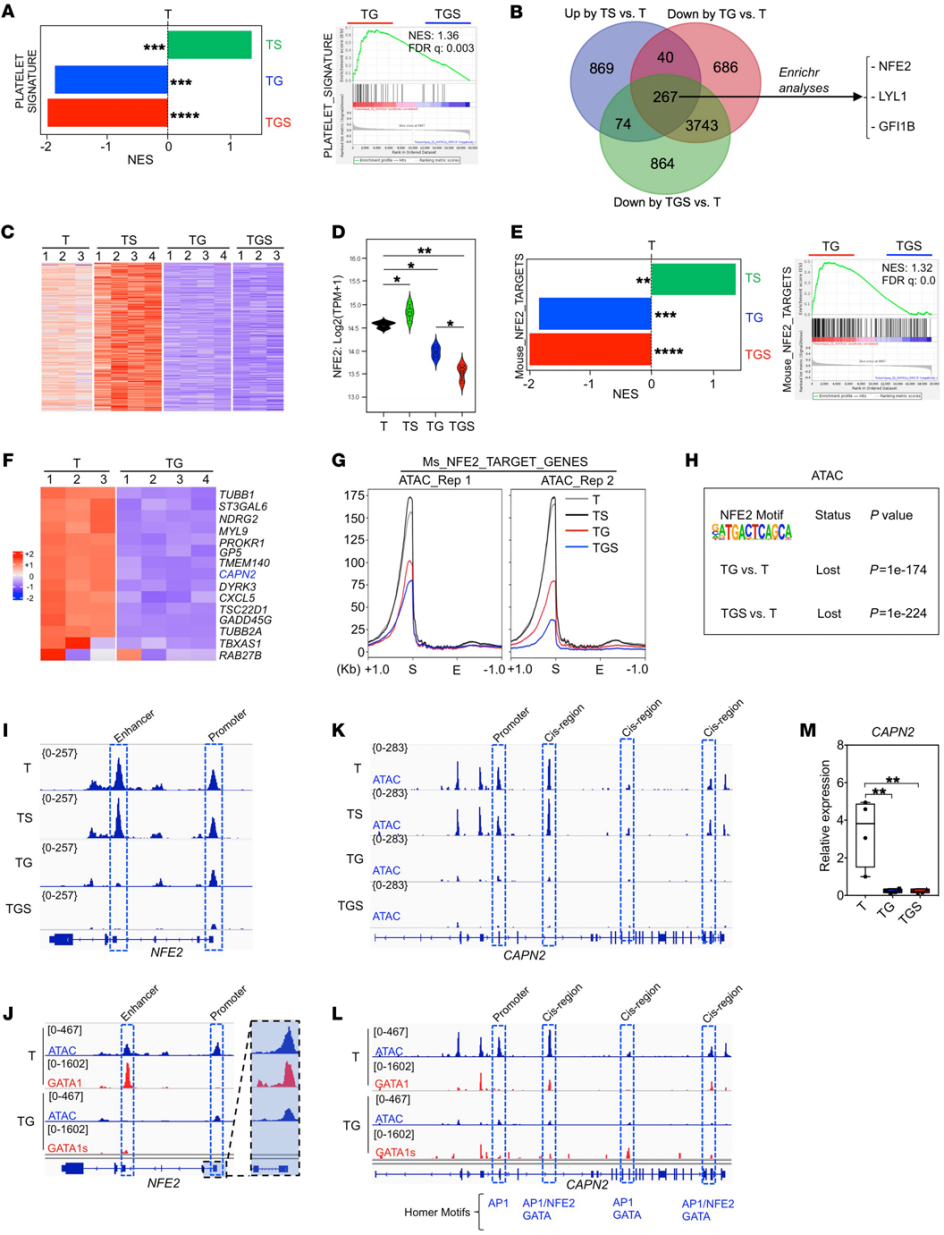
*GATA1s* cooperates with *SMC3^+/–^* to downregulate the NFE2 transcriptional program. (**A**) GSEA for platelet genes (47) in the indicated comparisons. ***FDR < 0.01; ****FDR < 0.001. (**B**) Venn diagram showing the overlap of up- and downregulated genes in the indicated comparisons. The top transcriptional signatures from the commonly downregulated genes in TG or TGS and upregulated in TS compared with T are annotated with an adjusted *P* < 0.0001 (Enrichr submissions TF-gene cooccurrence). (**C**) Heatmap showing the expression of the 267 genes that are common in all comparisons (up by TS versus T, down by TG versus T, down by TGS versus T) shown in the Venn diagram in **B**. (**D**) Violin plot showing *NFE2* expression in the indicated conditions. *P < 0.05 and *P < 0.01. (**E**) GSEA for NFE2 target genes (39) in TS versus T, TG versus T or TGS versus T (left panel), and TGS versus TG (right panel) iMKs. **FDR < 0.05; ***FDR < 0.01; ****FDR < 0.001. (**F**) Heatmap showing the expression of NFE2 target genes that are downregulated in TG compared with T. *CAPN2* gene marked in blue is also known as a direct target of GATA1. (**G**) Profile plots of ATAC signal from NFE2 target genes (Zang et al., 2016) comparing T, TS, TG, and TGS iMKs. The start (S) and the end (E) of the genes were plotted across a 1 kb flanking window; the *y* axis indicates depth per million mapped reads. Left: replicate 1; right, replicate 2. (**H**) Relative loss of the NFE2 motif in ATAC-Seq data from the indicated comparisons. (**I**) ATAC-Seq profile on *NFE2* in T, TS, TG, and TGS. (**J**) Track depicting CUT&TAG-Seq for GATA1 and GATA1s and ATAC-Seq on the *NFE2* locus in T or TG iMKs. (**K**) ATAC-Seq profile on *CAPN2* in T, TS, TG, and TGS. (**L**) Track depicting CUT&TAG-Seq for GATA1 and GATA1s and ATAC-Seq on the *CAPN2* locus in T or TG iMKs. (**M**) RT-qPCR analysis of *CAPN2* expression in T, TS, TG, and TGS iMK.

Since *SMC3* haploinsufficiency-induced PPT formation in GATA1^WT^ was abrogated in a GATA1s context (Figure 3, E and F), we searched for molecular signatures enriched in TS but depleted in TG and TGS contexts. This included platelet gene signatures (Figure 5A). Enrichment analysis of the 267 common genes suggested that the NFE2-regulated program was inhibited (Figure 5, B and C). Notably, while NFE2 is a well-characterized target of GATA1 in terminal MK differentiation (33), it is not deregulated by Gata1s in mouse MKs (34). Here, *NFE2* expression was profoundly downregulated in a stepwise manner (Figure 5D). GSEA and heatmap analysis showed a depletion in NFE2 targets in TG versus T MKs (Figure 5, E and F) and TGS versus TG MKs (Figure 5E). In contrast, *SMC3^+/–^* in a GATA1^WT^ (TS) showed an increased expression of both *NFE2* and NFE2 target genes (Figure 5, D and E), supporting the enhanced PPT formation.

ATAC-Seq profiles over TSS of NFE2 target genes showed a gradually lower accessibility in TG and TGS compared with either T or TS MKs (Figure 5G). DNA-binding motif analyses indicated that GATA1s led to a relative loss of chromatin accessibility at NFE2 (*P* = 1 × 10^–174^, Figure 5H) as well as of other AP1 family and GATA motifs (Supplemental Figure 7C) and gain in chromatin accessibility at RUNX and ETS motifs (Supplemental Figure 7D) in both TG and TGS (Supplemental Figure 7, E and F). The ETS and RUNX motifs as well as AP1 family motifs were further lost in TGS compared with TG (Supplemental Figure 7G), while no motif was significantly gained (Supplemental Figure 7H).

Importantly, there was a good correlation between *NFE2* gene expression and its chromatin accessibility with a marked decreased accessibility at its promoter as well as cis-regulatory enhancer by GATA1s (Figure 5I). The CUT&Tag approach (35) showed a direct binding of GATA1 at both the *NFE2* promoter and enhancer (T21 context) with a strong reduction in GATA1s binding (TG context; Figure 5J). Notably, a similar observation was made for *CAPN2* (Figure 5, K–M), which was previously demonstrated to be altered by Gata1s in mouse MK (12). Our integrative approach revealed that *NFE2* is a direct target of GATA1 in human MKs, and that GATA1s plus SMC3 mutation leads to a stepwise reduction in *NFE2* expression associated with a global inhibition of the NFE2-mediated transcriptional program.

### NFE2 is involved in the MK development defect induced by GATA1s.

**Figure 6:**
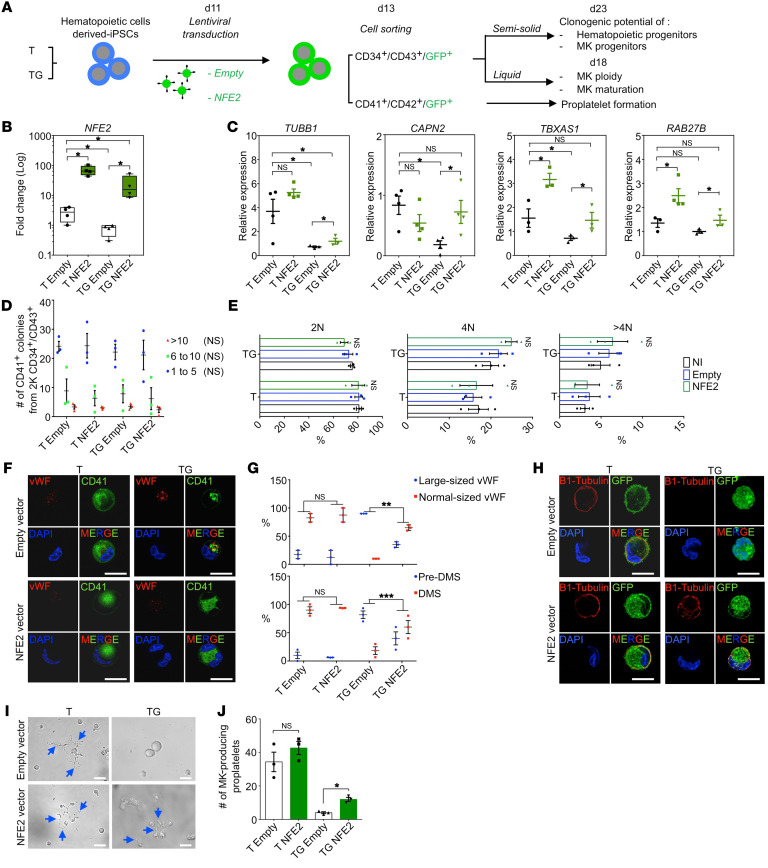
NFE2 overexpression in TG MK partially rescues maturation and PPT defects. (**A**) Schematic overview of NFE2 overexpression strategy in T and TG iMKs and of subsequent phenotypic studies. (**B**) Validation by RT-qPCR of NFE2 overexpression in T and TG iMKs. (**C**) RT-qPCR analyses of the known NFE2 target gene expression with empty or NFE2 lentiviral vectors. (**D**) Effect of NFE2 overexpression on the clonogenic potential of T and TG iMKs. (**E**) Effect of NFE2 overexpression on the ploidization of T and TG iMKs. (**F**) Confocal analyses of NFE2 overexpressing MK in T and TG for CD41 and vWF expression. Scale bars: 50 μm. (**G**) Histograms of the percentages of MKs with normal or large-sized vWF (upper panel) and the percentages of MKs with pre-DMS or DMS (lower panel) according to the absence (empty) or presence of NFE2 lentiviral vector. Quantifications were performed on 20 MKs from 3 independent experiments. (**H**) Confocal analyses of β_1_-tubulin expression in T- and TG-derived MKs according to the absence (empty) or presence of NFE2 lentiviral vector. Scale bars: 50 μm. (**I**) Representative microphotographs of CD41^+^CD42^+^ MKs under PPT formation assay. Note the presence of PPT-forming MKs (blue arrows) in the absence or presence of NFE2 lentivector for the T, while in TG, the presence of PPT-forming MK was observed only in the presence of NFE2 lentivector (blue arrows). Scale bars: 50 μm. (**J**) Histogram of the number of PPT-forming MK shown in **I** according to the compared genotypes. Data are represented as mean ± SEM; *n* = 3. Statistical significance was determined using 1-tailed Mann-Whitney’s *U* test. *P < 0.05; **P < 0.01; ***P < 0.001.

To assess the role of *NFE2* downregulation by GATA1s, we transduced empty or *NFE2*-encoding lentiviral vector into T and TG iPSC-derived hematopoietic cells. Transduced CD34^+^CD43^+^ HPCs were sorted according to GFP expression and induced into MK differentiation (Figure 6A). Ectopic *NFE2* was confirmed by quantitative reverse-transcriptase PCR (RT-qPCR) in T and TG (Figure 6B). Known NFE2 target genes that were strongly downregulated upon *GATA1s* acquisition were significantly upregulated in *NFE2*-transduced compared with empty vector–transduced TG MKs (Figure 6C). *NFE2* expression in TG cells completely restored levels of *CAPN2*, *TBXAS1*, and *RAB27B* and partially restored levels of *TUBB1*.

*NFE2* expression did not influence the clonogenic potential of either MK or HPC progenitors in T or TG (Figure 6D, Supplemental Figure 8, A and B), supporting the idea that NFE2 does not regulate MK progenitor proliferation. Also, *NFE2* reexpression had no effect on the ploidy (Figure 6E and Supplemental Figure 8C) or the percentages of CD41^+^CD42^+^ and CD34^+^ cells (Supplemental Figure 8, D–F). However, NFE2 expression significantly decreased the proportion of large megakaryoblasts with abnormal α-granules and a pre-DMS, the maturation signature defect of TG MKs (Figure 6, F and G). Furthermore, ectopic NFE2 induced expression of β_1_-tubulin located along the cytoplasmic membrane in 30% of TG MKs (Figure 6H) and partly rescued PPT formation by TG MK (Figure 6, I and J).

### Contribution of the T21 to the cooperation between GATA1s and SMC3^+/–^.

To characterize the contribution of T21 in the phenotype described above, we generated an isogenic DGM clone and further introduced the SM*C3^+/–^* mutation to obtain DGMS clones (Figure 1A and Supplemental Figure 1, T and V).

**Figure 7:**
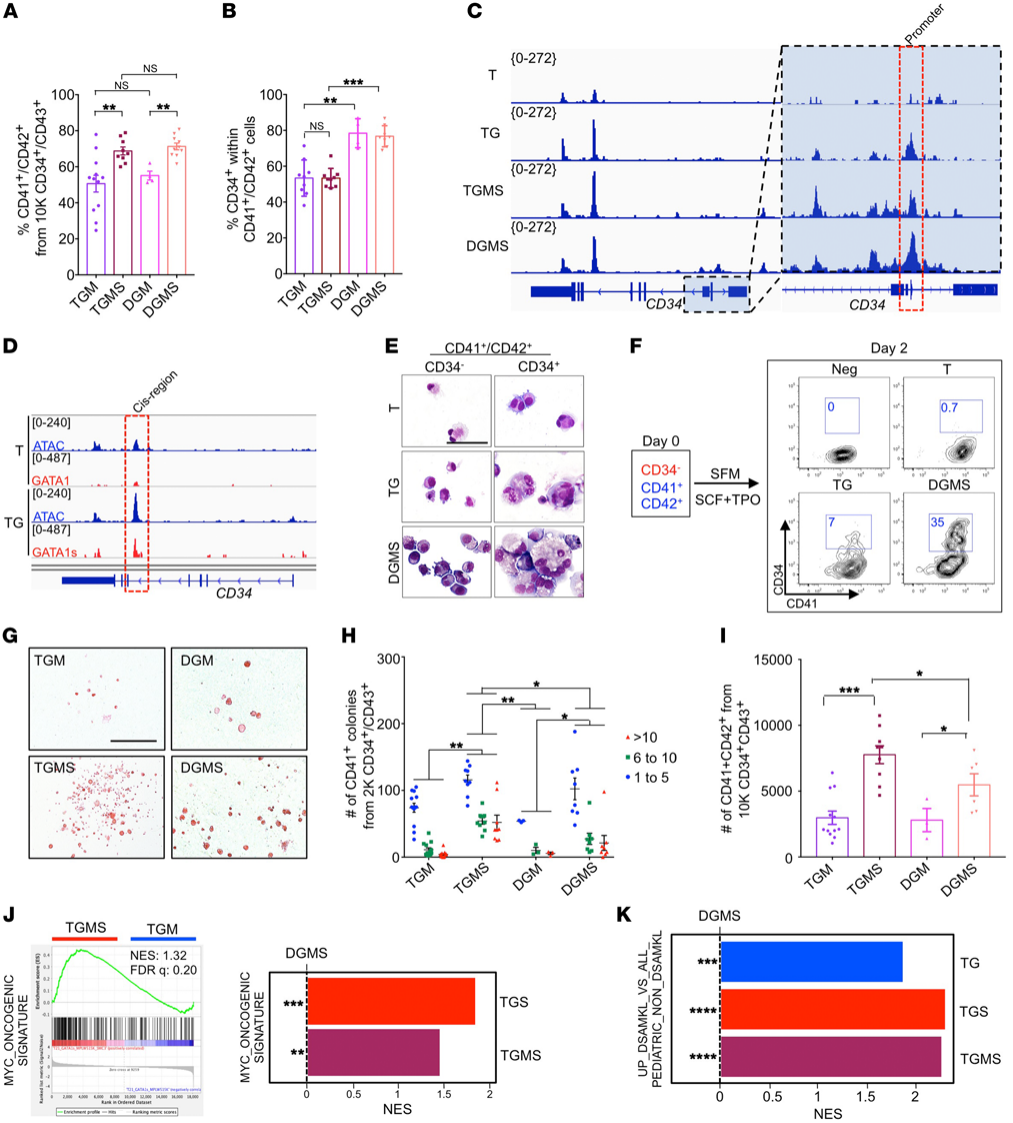
T21 acts in synergy with GATA1s and *SMC3^+/–^* to increase MK proliferation and to acquire DS-AMKL features. (**A**) Histogram of the mean percentage of CD41^+^CD42^+^ cells. (**B**) Percentage of CD34^+^CD41^+^ within the CD41^+^ MK population. Data are represented as mean ± SEM; *n* = 3 to 4. The number of clones tested per genotype was as follows: TGM = 4; TGMS = 3; DGM = 1; DGMS = 2. (**C**) ATAC-Seq profile on *CD34* in T, TG, TGMS, and DGMS. (**D**) Track depicting CUT&TAG-Seq for GATA1 and GATA1s and ATAC-Seq at the *CD34* locus in T or TG iMKs. (**E**) May-Grünwald-Giemsa coloration of CD34^–^ or CD34^+^ fractions within the CD41^+^CD42^+^ cell population in T, TG, and DGMS. Scale bar: 50 μm. (**F**) Contour plots show CD34 expression in T, TG, and DGMS iMKs after 2 days of culture. Negative control (Neg CTL): MKs incubated without CD34 antibody. (**G**) Representative microphotographs of CFU-MK colonies in fibrin clot assay for TGM, TGMS, DGM, and DGMS. Scale bar: 500 μm. (**H**) Mean number of CFU-MK colonies obtained from 2000 CD34^+^CD43^+^ in fibrin clot assay. (**I**) Mean number of CD41^+^CD42^+^ MKs from 10,000 CD34^+^CD43^+^ in liquid cultures. (**J**) GSEA for the *MYC* oncogenic signature comparing indicated conditions. (**K**) GSEA for the upregulated genes in DS-AMKL versus non-DS AMKL pediatric patients in TGS versus DGMS or TGMS versus DGMS iMKs. Statistical significance was determined using 1-tailed Mann-Whitney *U* test. *P < 0.05; **P < 0.01; ***P < 0.001.

The percentage of CD41^+^CD42^+^ MKs generated in isogenic D and T conditions was close and above 50% (Supplemental Figure 9A and Figure 7A). Unexpectedly, the percentage of CD34^+^CD41^+^ cells was significantly higher in isogenic clones, nearly 80% of the CD41^+^ cells reproducing the differences previously observed between T21 and D21 MKs (Supplemental Figure 3C, Supplemental Figure 9A, and Figure 7B). This was correlated with a higher chromatin accessibility at the CD34 promoter in TG and TGMS versus T, which was even higher in DGMS (Figure 7C). CUT&Tag for GATA1 revealed a higher binding of GATA1s compared with GATA1 at a cis-intronic region of *CD34* (Figure 7D). At the cellular level, the CD34^–^ fraction of CD41^+^CD42^+^ MKs in TG or DGMS corresponded to small immature blast cells while the CD34^+^ fraction included the large polyploid megakaryoblasts (Figure 7E). Therefore, we hypothesized that CD34 expression is aberrantly upregulated upon differentiation in GATA1s MKs. To verify this hypothesis, CD34^–^CD41^+^CD42^+^ MKs from T, TG, and DGMS were sorted and seeded with SCF and TPO to investigate phenotypes after 2 days of culture (Figure 7F). While T MKs remained CD34^–^CD41^+^CD42^+^, some TG MKs, and even more so DGMS MKs, reacquired CD34 expression (Figure 7F). These results support the idea that aberrant reacquisition of CD34 expression is controlled by GATA1s in both T and D MKs, with a higher expression in the latter context. Notably, there was no difference in the ploidy between T and D, with a similar increase in the mean percentage of more than 4N MKs by *SMC3^+/–^* (Supplemental Figure 9B). MK morphological abnormalities, including α-granules and DMS, were identical at confocal and electron microscopy for DGMS and TGMS MKs (Supplemental Figure 9, C and D). PPT formation was similarly abrogated in DGM and DGMS versus TGM and TGMS (Supplemental Figure 9, E and F). The lack of significant enrichment of platelet signature gene lists further supported the proximity between DGMS and TGMS MK maturation defects (Supplemental Figure 9G). Therefore, the data show that T21 is not directly involved in the MK maturation defect induced by GATA1s and its cooperation with *SMC3*^+/–^.

We subsequently studied whether T21 may affect HPC proliferation. DGM and TGM CD34^+^CD43^+^ cells showed similar MK clonogenic potential (Figure 7, G and H). In contrast, TGMS HPCs had higher MK clonogenic potential than their DGMS counterparts, with a higher proliferative capacity highlighted by an increased number of MK colonies composed of more than 10 cells and by an increased MK yield in liquid cultures (Figure 7, G–I). Of note, some rare very large TGMS colonies composed of more than 1000 MKs could be observed.

Differential gene expression analyses revealed 495 up- and 289 downregulated genes in TGMS versus DGMS (Supplemental Figure 6, G and H). More specifically, GSEA showed enrichment of a MYC signature in both TGS and TGMS versus DGMS MKs as well as TGMS versus TGM MKs (Figure 7J), indicating that *SMC3^+/–^* cooperated with T21 and *GATA1s* to enhance the MYC-induced proliferation program. Importantly, GSEA with a DS-AMKL gene list showed signatures significantly enriched in TGMS versus DGMS iMKs (Figure 7K). Among the genes enriched in TGMS versus DGMS and in TG versus DGMS MKs was MYB (Supplemental Figure 9, H and I), a regulator of myeloid leukemogenesis (36, 37). scRNA-Seq between DGMS and TGMS showed that T21 increased MK output as well as all MK-related clusters, including the abnormal cluster 20 (Figure 2, I–K). Together, the results indicate that T21 contributes to the molecular signature of DS-AMKL and cooperates with GATA1s and *SMC3^+/–^* in part through positive regulation of a MYC and MYB proliferative program.

### Pharmacological inhibition of MYC reduces megakaryoblast expansion and enhances their differentiation.

**Figure 8:**
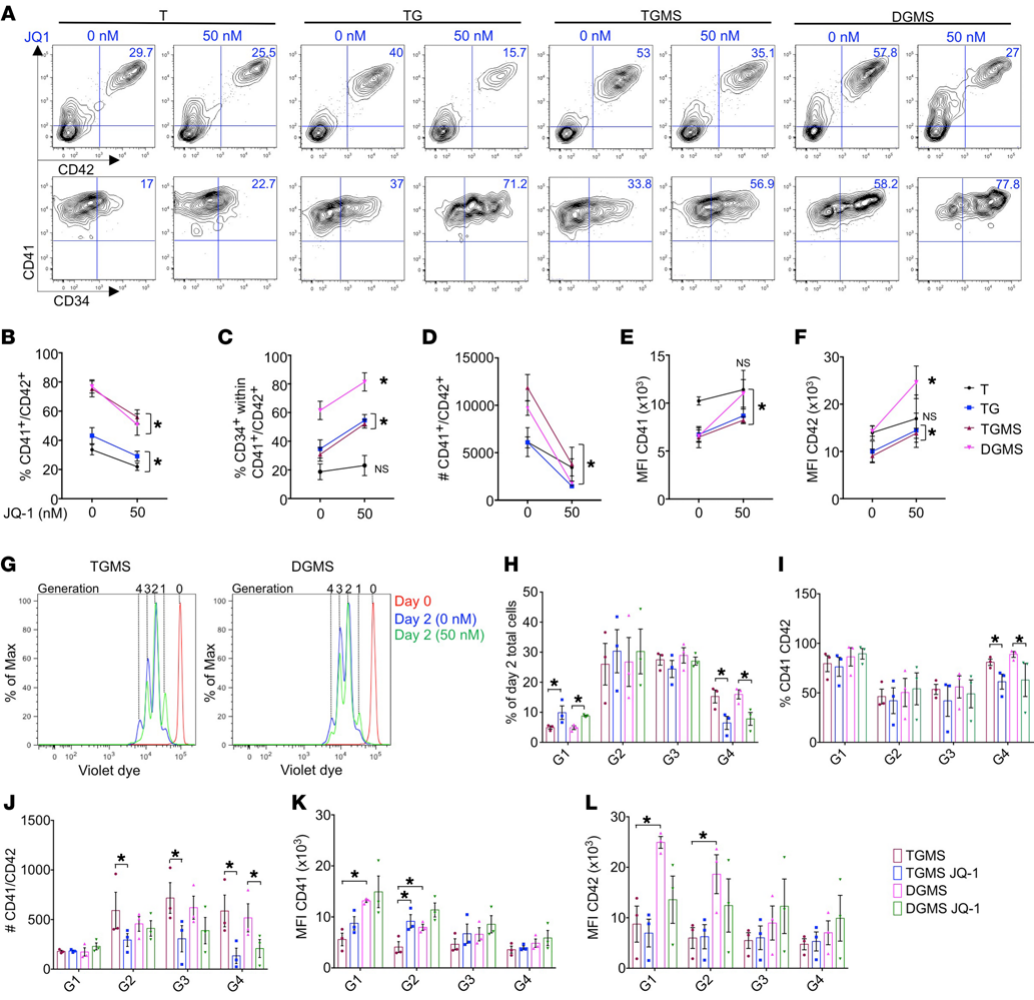
Assessment of MYC inhibition on iMK proliferation and differentiation. (**A**–**F**) Immunophenotypes of iMKs for the CD34, CD41, and CD42 markers found after JQ1 treatment. (**A**) Contour plots showing the expression of CD34, CD41, and CD42 markers. (**B**) Before-and-after graph shows the percentage of CD41^+^ CD42^+^. (**C**) Before-and-after graph shows the percentage of CD34^+^ cells within the total CD41^+^CD42^+^ cell population. (**D**) Before-and-after graph shows the absolute number of CD41 and CD42. (**E**) Before-and-after graph shows the MFI of CD41. (**F**) Before-and-after graph shows the MFI of CD42. Data in **B** through **F** are represented as mean ± SEM; *n* = 3. Conditions with or without JQ-1 are compared, and statistical significance was determined using 1-tailed Mann-Whitney’s *U* test. *P < 0.05. (**G**) Representative flow cytometry plots of a violet dye experiment at day 2 of culture in the TGMS versus DGMS. Red, day 0 of culture; blue, dimethyl sulfoxide control; green, JQ-1. The number of generations is indicated at the top of the plot. (**H**) Histogram of the percentage of day 2 total cells per generation (G) in the indicated conditions. (**I**) Histogram shows the percentage of CD41^+^CD42^+^ per generation in the indicated conditions. (**J**) Histogram shows the absolute number of CD41^+^CD42^+^ in the indicated conditions per generation. (**K**) Histogram shows the MFI of CD41 per generation in the indicated conditions. (**L**) Histogram shows the MFI of CD42 per generation in the indicated conditions. Data are represented as mean ± SEM; *n* = 3. Conditions with or without JQ-1 are compared, and statistical significance was determined using the Kruskal-Wallis test. *P < 0.05.

To investigate whether MYC inhibition can target the proliferating megakaryoblasts, hematopoietic progenitors from T, TG, TGMS, and DGMS were treated with JQ-1 inhibitor in a dose-dependent manner for 5 days (Supplemental Figure 10, A–E). JQ-1 decreased the percentage of CD41^+^CD42^+^ cells, while it increased the percentage of CD34^+^ cell fraction within T, TG, TGMS, and DGMS MK populations as compared with the nontreated conditions (Figure 8, A–C). Notably, even very low concentration of JQ-1 (50 nM) dramatically decreased the absolute number of TG but also TGMS or DGMS megakaryoblasts (Figure 8D). In parallel, JQ-1 restored the expression levels of CD41 and CD42 in TG, TGMS, and even more in DGMS compared with the respective nontreated conditions (Figure 8, E and F). To precisely determine the impact of JQ-1 inhibitor on proliferation and differentiation, we evaluated the expression of CD41 and CD42 according to the number of divisions using a violet dye cell tracker after 2 days of treatment (Figure 8G). JQ-1 significantly decreased the percentage of total cells, but also the percentage of CD41^+^CD42^+^ cells at division 4 in TGMS and DGMS as compared with the nontreated conditions (Figure 8, H and I). Importantly, JQ-1 induced a dramatic decrease in the absolute number of TGMS MK cells from division 2 until division 4 and even more marked in TGMS than DGMS (Figure 8J). Furthermore, JQ-1 did not yet influence the MFI of CD42 at the day 2 early time point whatever the number of divisions. However, it increased the MFI of CD41 through division 2 in TGMS at a level close to that observed in the nontreated DGMS (Figure 8, K and L). These results show that JQ1 has a much more rapid and significant effect in TGMS than in DGMS.

Together, these results strongly suggest that the MYC transcriptional program plays a key role in acquiring the leukemic features of DS megakaryoblasts and can be pharmacologically targeted.

## Discussion

We modeled the defect in MK differentiation observed in human DS-AMKL through stepwise introduction of *GATA1* (*GATA1s*), MPL (*MPL^W515K^*), and *SMC3* haploinsufficiency (*SMC3^+/–^*) mutations in T21 iPSCs to characterize the contribution of individual alterations and how they cooperate to alter hematopoiesis and impose MK differentiation blockage. We showed that GATA1s impairs MK differentiation and that *SMC3^+/–^* enhances this phenotype by synergistically acting with GATA1s to induce an even more profound failure of the GATA1-dependent MK differentiation program. These differentiation alterations were independent of T21, supporting the recent observation that T21 is not mandatory for inducing DS-AMKL by the combined *GATA1* and *STAG2* mutations (19). However, T21 cooperates with *SMC3^+/–^* to increase proliferation. Also, *MPL*-activating mutation further enhanced MK output, including through induction of growth factor independence, but did not affect differentiation, as seen with *FLT3* mutation in another iPSC model of AML (38).

These data uncovered the importance of using human cells for the development of DS-AMKL models. Indeed, murine Gata1s models revealed increased proliferation of yolk sac and fetal liver MKs with minimal differentiation alteration and the generation of mature PPT-forming MKs (10). In contrast, the present study showed that GATA1s markedly affects the MK differentiation of both D21 and T21 human iPSCs, inducing the emergence of megakaryoblasts with major impairments in DMS, α-granules, and platelet formation.

Molecularly, numerous GATA1 target genes involved in MK differentiation were downregulated in GATA1s MKs (e.g., *GPIBA*, *PACSIN2*, *RAB* genes and *TUBB1*, respectively, involved in DMS, granule, and PPT formation). Previously, only 2 GATA1 targets (*GPIBA* and *CAPN2*) were reported as downregulated in *Gata1s* murine models (34, 12). We found that NFE2 and its transcriptional program were profoundly inhibited in human iMKs, which is consistent with the essential role of NFE2 in the regulation of late MK differentiation (33, 39, 40). This resulted from a lower binding of GATA1s to *NFE2* regulatory elements and from further reduction of chromatin accessibility at *NFE2* and NFE2 target loci upon addition of *SMC3^+/–^* with a concomitant gain in accessibility at ETS motifs (e.g., FLI1 and ERG). Interestingly, *SMC3^+/–^* has the opposite effect in a GATA1^WT^ background by promoting MK differentiation with an increased PPT formation associated with an enhanced expression of genes involved in MK terminal differentiation, including an NFE2 signature. Therefore, this reveals a functional interplay between cohesin and GATA1 that is dependent of the chromatin organization shaped by GATA1 or GATA1s. The positive effect of *SMC3^+/–^* on GATA1^WT^ MK maturation was unexpected, as it was reported that a cohesin defect blocks terminal erythroid and myeloid differentiation. However, the precise effect of cohesin mutations on MK maturation was not previously studied, apart from the report of small hypoploid MKs in *Stag2*-deficient mice (24). Together with the functional rescue of PPT formation upon NFE2 ectopic expression, these results emphasized the key role of NFE2 in GATA1s-induced MK differentiation blockage that is further enhanced upon cooperation between *GATA1s* and *SMC3^+/–^*. Thus, the MK differentiation defect induced by GATA1s results from the altered expression of (a) genes directly regulated by GATA1 and (b) genes indirectly regulated through NFE2. Although murine models of Gata1s in combination with T21 and cohesin mutation have not been reported to date, the differential impact of GATA1s on *NFE2* expression in humans and mice may represent a molecular basis for incomplete DS-AMKL modeling in mice.

The predominant effect of endogenous *Gata1s* on mouse hematopoiesis is the induction of hyperproliferation of MK progenitors (10). In this study, we did not observe a significant increase in the number and proliferation of MK progenitors nor on the output of MKs defined as CD41^+^CD42^+^ cells upon engineering of GATA1s. This is consistent with the effect of *Gata1s* on murine ES cells (13) and the recent observation showing that the increased MK output generated by GATA1s is due to a shift in HSC differentiation toward the MK lineage rather than to a direct effect on MK progenitors (18, 19). However, our results showed a marked synergy between *SMC3^+/–^* and GATA1s on both proliferation (e.g., increased MK progenitor number and size of MK colonies) and ploidization. This dual effect could be explained by the enrichment in a MYC program when combining T21, GATA1s, and SMC3*^+/–^*, also seen in other models of *Smc3* haploinsufficiency (41). Indeed, a requirement for a transient MYC activity was reported for MK ploidization (42, 43), while its sustained activity is associated with impaired maturation (42). Notably, the *SMC3^+/–^*-dependent gain in RUNX1/ETS binding site accessibility in T21 MKs may synergize with the previously reported loss of interaction between RB1 and GATA1s (44). An increased level of MYC and of MYC activity in DS-AMKL in comparison with other pediatric AMKL has been previously reported (45), and recently it has been reported that it is related to an excess of RUNX1A isoform (46). Here, we showed that low-dose BET inhibitor treatment inhibits both proliferation and survival of megakaryoblasts. Our data suggest that the MYC pathway is a major and targetable pathway in DS-AMKL and are consistent with a recent study showing that MYC inhibition induces apoptosis of DS-AMKL blasts (46).

While allowing reproducible cellular and molecular analyses from the same clone, the current protocol used to obtain iPSC-derived hematopoiesis does not recapitulate a definitive hematopoiesis with apparent lack of true HSC. This precludes efficient engraftment into immunodeficient mice and clearly represents a limitation in our current model. Indeed, *SMC3* haploinsufficiency did not induce a strong in vitro self-renewal of HSPC nor the engraftment into NSG immunodeficient mice (data not shown) as opposed to what is seen with human primary fetal *GATA1s* *STAG2*^KO^ HSPCs (19). Successful engraftment of hematopoietic cells derived from AML or high-grade MDS-iPSC or using an approach similar to ours showed that the sequential introduction of mutations involved in AML (*ASXL1*, *SRSF2*, *NRAS*) allowed low burden in vivo engraftment, but without the capacity of serial transplantations (38). Whether bona fide leukemic engraftment of an iPSC model of DS-AMKL will require more drastic consequences on HSPC self-renewal than *SMC3^+/–^*, such as mutation of *STAG2* presenting only one transcribed allele located on the active X chromosome or a fourth alteration (e.g. targeting the PRC2 complex) (8, 9) or different culture or recipient conditions, remains to be determined. This aspect clearly represents a limitation in our modelization. Nonetheless, such a stepwise introduction of driver mutations of DS-AMKL reproduces the MK leukemic differentiation blockage, sheds light on its molecular mechanism, and paves the way for future studies on other leukemogenesis processes necessary for inducing self-renewal.

In summary, the present in-depth cellular and molecular characterizations of the iPSC-based model contribute to better understanding the progression from TMD to AMKL seen in children with Down syndrome. Specifically, we demonstrated that the low expression of *NFE2* is critical for the induction of MK dysplasia by GATA1s, uncovering a molecular basis of the cooperation among cohesin mutation, GATA1s, and T21 in the MK differentiation defect of DS-AMKL.

## Methods

Further information can be found in the Supplemental Methods.

### iPSC generation and expansion.

The trisomic 21 iPSC line (T21.2; 47XY, +21) was a gift from Stella Chou and Mitchell Weiss. It was derived as previously described (14) through reprogramming of fetal stromal cells after transduction with pMXs-based retroviral supernatant encoding human OCT4, SOX2, KLF4, or MYC. iPSCs were maintained in Essential 8 or Essential 8 Flex Medium (Gibco; Thermo Fisher Scientific) on plates coated with N-truncated human recombinant vitronectin (Gibco; Thermo Fisher Scientific). Cell passages were performed mechanically or by using the StemPro Accutase Solution (Gibco; Thermo Fisher Scientific). A mycoplasma screening was routinely performed, according to the manufacturer’s instructions (MilliporeSigma). A list of manufacturers with the catalog number of each product is provided in Supplemental Table 2.

### iPSC genome editing.

In order to obtain *GATA1s* mutation, oligo encoding sgRNA targeting exon 2 of the gene was inserted into pX330 GFP plasmid (Addgene), while for *SMC3^+/–^* mutation, sgRNA targeting exon 9 was inserted into lenti-CRISPR V2 GFP plasmid (provided in house). For each experiment, 5 μg of GFP-expressing vector was electroporated into 1 × 10^6^ iPSCs with Human Stem Cell Nucleofector Kit 1 (Lonza) following the manufacturer’s instructions. Seventy-two hours later, the GFP-expressing cells were sorted by FACS and seeded at low density. From 9 to 11 days after seeding, 10 or 40 individual clones were picked manually for *GATA1s* or *SMC3^+/–^*, respectively, where two-thirds of each colony were reserved for genomic DNA extraction. For *GATA1s*, DNA was amplified by PCR using the primers listed in Supplemental Table 3. *GATA1* PCR amplicons were submitted to Sanger sequencing (Eurofins), and 2 clones harboring a frame shift with a premature stop codon in exon 2 were selected. For *SMC3^+/–^* clone genotyping, 500 ng of purified PCR amplicons were incubated with BanII restriction enzyme (NEB) for 2 hours at 37°C. The genotype was determined thereafter by 2% agarose gel migration. To identify *SMC3* indels of heterozygous mutated clones, the nondigested band was submitted to Sanger sequencing (Eurofins).

For *MPL^W515K/W515K^* knockin, an sgRNA with a PAM corresponding to W515 of the *MPL* gene was selected and cloned into the lenti-CRISPR V2 GFP. The 5′ and 3′ homology arms of 700 bp of *MPL* surrounding a PGK cherry selection cassette were cloned into pUC57 vector (GenScript). Both CRISPR and donor vectors were cotransfected at a ratio of 2 μg:6 μg, respectively, into 3 × 10^6^ iPSCs using the Human Stem Cell Nucleofector Kit 1 (Lonza). Six days later, cherry-positive expression selection by FACS was performed for clonal expansion. The genotypes of 12 cherry-positive clones were determined by PCR screening of genomic DNA and validated by Sanger sequencing (Eurofins). To delete the PGK-cherry cassette, correctly targeted clones were transfected with flippase-expressing plasmid, and 48 hours later, cells without cherry expression were sorted and expanded. PGK cherry cassette excision was confirmed by PCR.

The guide RNA design and off-target identification were determined using CRISPOR software (http://crispor.tefor.net). Potential indels were screened considering 1 to 4 mismatches by PCR/sequencing. For each edited gene, off-target loci containing a CFD score greater than 0.1 were selected. Off-target regions of each sgRNA used have been amplified by PCR and verified by Sanger sequencing (Eurofins). Karyotyping analyses of all targeted clones were performed by G-banding in a cytogenetics facility (Hôpital Antoine Béclère). All oligos of the different sgRNAs targeting GATA1, SMC3, and MPL genes and those used for PCR amplification are listed in Supplemental Table 3.

### Hematopoietic differentiation from iPSC clones.

Clumps from iPSC colonies were seeded on Geltrex-coated plates in Essential 8 medium at day −1. The starting cell concentration was at a 20% confluency range that corresponded to approximately 500 to 1000 single cells from 1 iPSC clone per experiment. At day 0, StemPro-34 SFM (Gibco; Thermo Fisher Scientific) xeno-free medium was used, supplemented with 1% penicillin/streptomycin (v/v; Gibco; Thermo Fisher Scientific), 1% l-glutamine (v/v; Gibco), 0.04 mg/mL of 1-thioglycerol (MilliporeSigma), and 50 mg/mL of ascorbic acid (MilliporeSigma). This medium was retained until day 13 and supplemented with different small molecules, cytokines, and growth factors in a time-dependent manner, as described in Supplemental Table 4. At day 13, CD34^+^CD43^+^ HPC-like cells of the supernatant were sorted (see Supplemental Methods) and seeded to undergo MK differentiation in a serum-free medium containing 25 ng/ml hSCF (Biovitrum) and 25 ng/ml hTPO (Kirin Brewery). Functional validation experiments of the *MPL^W515K/W515K^* were performed by adding or not adding 1 μM ruxolitinib (Euromedex). All incubations were performed at 37°C, 5% CO_2_. A list of manufacturers with the catalog number of each product is provided in Supplemental Table 2.

### Statistics.

Statistical analyses were performed with GraphPad Prism software 70.a. Comparisons between different groups were performed using a 1-tailed unpaired Mann-Whitney *U* test or 1-way ANOVA Kruskal-Wallis test. Data are represented as mean ± SEM. For all analyses, *P* < 0.05 was considered statistically significant.

### Data access.

RNA-Seq, scRNA-Seq, ATAC-Seq, and CUT&Tag raw data were deposited in ArrayExpress (E-MTAB-11032, E-MTAB-11033, E-MTAB11036, E-MTAB-11044).

## Author contributions

BA, HR, TM, and WV designed the study. BA, FB, S Mazzi, VD, AS, YM, ZA, MV, IA, MC, N Debili, and N Droin performed gene editing and cellular and molecular analyses. SS performed electron microscopy. RP, PR, CC, RD, GT, OH, IP, S Malinge, and ES contributed essential reagents and expertise. ER, FB, and MA performed the bioinformatics analyses with help from BA, FB, TM, and WV. BA, TM, and WV drafted the manuscript. All authors approved the final manuscript.

## Supplementary Material

Supplemental data

## Version 1 05/19/2022

In-Press Preview

## Version 2 07/15/2022

Electronic publication

## Version 3 04/26/2023

Updated author list
