# Supplementary material for: Stepwise GATA1 and SMC3 mutations alter megakaryocyte differentiation in a Down syndrome leukemia model
Source: J Clin Invest. 2022 Jul 15;132(14):e156290. doi: 10.1172/JCI156290 (PMC9282925; doi:10.1172/JCI156290)

Stepwise GATA1 and SMC3 mutations alter megakaryocyte differentiation in a Down syndrome leukemia model

Brahim Arkoun[1,2,3], Elie Robert[3], Fabien Boudia[3], Stefania Mazzi[1], Virginie Dufour[4], Aurélie Siret[3], Yasmine Mammasse[4], Zakia Aid[3], Matthieu Vieira[1], Imanci Aygun[1], Marine Aglave[5], Marie Cambot[4], Rachel Petermann[4], Sylvie Souquere[6], Philippe Rameau[7], Cyril Catelain[7], Romain Diot[8], Gérard Tachdjian[8], Olivier Hermine[2,9], Nathalie Droin[1,10], Najet Debili[1], Isabelle Plo[1,2], Sébastien Malinge[3,11], Eric Soler[2,12], Hana Raslova[1], Thomas Mercher[3,*], William Vainchenker[1,2,*]

Supplemental Data

Supplemental Methods

Cytospin preparation and cytologic staining

Sorted cell populations were centrifuged onto slides at 300*g* with Cytospin II (Thermo Fisher) for 5 minutes. Smears were stained with May-Grünwald-Giemsa.

Flow cytometry and cell sorting

The cells were resuspended in a serum-free medium containing 3% (v/v) Bovine Serum Albumin (Sigma). All antibodies were from BD Biosciences and are listed in Table S5. The cells were assayed on BD Fortessa and data were analyzed with FlowJo software. Cell sorting was performed on BD Influx or BD FACSAria Fusion.

Progenitors clonogenic assay

CD34 and CD43 double positive population was seeded at a concentration of 2000 cells/ml in MethoCult™ H4434 Classic (Stemcell Technologies, Grenoble, France) for 14 days to determine clonogenic potential of erythroid and myeloid progenitors. To quantify colony-forming-unit-megakaryocyte progenitors (CFU-MK), $CD34^+$ and $CD43^+$ cells were seeded at 2000 cells/ml in serum-free fibrin clots for 10 days. All experiments were performed in triplicates. MK progenitors were stained with an anti-CD41a monoclonal antibody (Fisher scientific; see Table S5) as previously described (Klimchenko *et al.*, 2009). Images were obtained using AxioVision 4.6 software.

Proliferation assay

$CD34^+$ and $CD43^+$ cells were seeded for 5 days under megakaryocytic culture condition (hSCF and hTPO) at a concentration of 100 000/ml in a 96 well plate. $CD41^+$ and $CD42^+$ megakaryocytes were counted using BD Fortessa (BD Biosciences).

Ploidy

DNA staining was performed by adding Hoechst (Thermo Fisher) staining solution to culture at a concentration of 10 µg/ml for 1 hour at 37 °C, 5% CO_2. Cells were then washed and resuspended in the same staining medium (PBS 1X with Hoechst 10 µg/ml) and suspended in staining medium. CD41a-APC and CD42a-PE antibodies (see Table S5) were used for BD Fortessa analyses.

Electron microscopy

Day 18 fresh megakaryocytes were fixed in 2% glutaraldehyde in 0.1 M phosphate buffer, pH 7.3 for 1 hour. Cells were pelleted and dehydrated in increasing concentrations of ethanol and embedded in Epon 812 according to routine procedures. Polymerization was carried out for 48 hours at 64°C. Ultrathin sections (90 nm) were obtained using an ultramicrotome (Leica) and stained with standard uranyl acetate and lead citrate and observed with FEI Tecnai 12 electron microscope. Digital images were taken with a SIS MegaviewIII CCD camera. The major criteria for classifying megakaryocytes into different stages are the quality and quantity of cytoplasm and the size, lobulation, and chromatin pattern of the nucleus. The immature megakaryocyte (megakaryoblast) is characterized by a large round, indented, or bilobed nucleus, prominent nucleoli and cytoplasm devoid of alpha granules with rudiments of the demarcation membrane system (DMS). The mature megakaryocyte is identified by its condensed nuclei containing eu- and heterochromatin, a cytoplasm with apha-granules and a well-developed DMS. 10 to 15 megakaryocytes from each condition were analyzed.

Immunofluorescence and confocal imaging

The cells were adhered onto polylysine slides for 1 h at 37°. Then, the cells were fixed with 4% paraformaldehyde for 10 min and permeabilized with a buffer containing 0.2% triton in PBS 1X for 10 min, and blocked for 30 min in a buffer containing 0.1% BSA in PBS 1X. Primary and secondary antibodies were diluted in PBS containing 0.1% BSA; incubation was performed for 1 h for each antibody. Primary antibodies: rabbit anti-vWF at 1:400 (Dako, A0082), mouse anti-CD41 at 1:100 (BD, clone HIP8), mouse anti-beta1-tubulin (abcam, ab204947), mouse anti-CD63 at 1:100 (Sigma, SAB4700215, clone MEM 259) rabbit anti-GFP (abcam, ab183734), secondary antibodies: goat anti-rabbit Alexa Fluor 546, goat anti-mouse Alexa Fluor 488 (Molecular Probes) both diluted at 1:500. Slides were mounted using Vectashield with DAPI (Molecular Probes). Images were acquired under a confocal Leica SP8 microscope, with a 63x/1.4 numeric aperture oil objective (Leica Microsystem). Image analysis and quantification of colocalization were performed with the LASX software.

Proplatelet formation

MK were sorted and seeded at a concentration of 30 000 cells/mL in 96 well plate in triplicate in presence of recombinant hSCF and hTPO for 3 days. Proplatelet formation was assessed by counting the number of MK producing proplatelet branches per well.

Retroviral particles production and transduction

For lentiviral transduction, we used either the pRRL-GFP (addgene) as a negative control, or the pLeGO-iG-GFP vector encoding the human *NFE2* open reading frame (kindly provided by Heike Pahl). Briefly, Lentiviral particles were produced in 293T cells which were co-transfected with plasmids of interest, along with a packaging plasmid (pCMV) and a VSV envelope expression plasmid (pMD2.G) using jetPRIME transfection reagent (Polyplus transfection, Ozyme). Supernatants were collected at 48h and concentrated by ultra-centrifugation.

RNA extraction and qRT-PCR

mRNA was isolated using a Direct-zol RNA MicroPrep Kit Kit (Zymo research) and quantified using a NanoDrop (Thermo Fisher Scientific). Reverse transcription was carried out with Vilo SuperScript Enzyme (Thermo Fisher Scientific). qPCR was performed with ONEGreen FAST qPCR Premix or Taqman Gene Expression Master Mix (Applied Biosystems) using a 7500HT Fast Real-Time PCR System (Applied Biosystems) according to the manufacturer's recommendations. The primer sequences are provided in Table S6.

Western blot

Total cells lysates were prepared as follows in 50mM Tris-HCl, pH 8, 150mM NaCl, 1% NP40, 1mM EDTA, 0,1% SDS, and 0,5% DOC supplemented with protease inhibitors (PMSF 1X, NAF 1X, Sodium Orthovanadate 1X, complete 1X). Western blotting was performed using standard procedures using the following antibodies from: anti-GATA1, anti-SMC3, anti-H3, anti-PCNA (Table S5).

GATA1/GATA1s quantification with flow cytometry

CD41+/CD42+ MK were prepared using the Cell Signaling Buffer Set A Kit (Miltenyi, 130-100-827). Cells were resuspended with Insid Fix solution and incubated for 10 min at RT and permeabilized using Permeabilization Buffer A at 4°C for 30 min. Thereafter, the cells were incubated with anti-GATA1/GATA1s antibody and then with Alexa 546-conjugated antibody (Table S5) at RT for 30 min. All washing steps were performed with PBS containing 0.5% BSA.

MYC inhibition with JQ-1

Isolated CD34$^+$ CD43$^+$ cells were cultured in serum-free medium in presence of recombinant human thrombopoietin (TPO; Kirin Brewery, Tokyo, Japan) and stem cell factor (SCF; Biovitrum, Stockolm, Sweden). MYC inhibition was achieved using JQ-1 (Clinisciences) in a dose dependent manner varying from 10 to 100 nM on day 0 and then day 2. At day 5 of culture, the cells were stained with CD41a-APC and anti-CD42a-PE antibodies before they were analased using BD Fortessa.

Violet dye

Hematopoietic progenitors derived from iPS cells were first stained using CD34-PE-Cy7 and CD43-FITC antibodies (BD Pharmingen). Violet dye (BD Horizon) was then added following manufacturer's protocol. Cells positive for both markers were then sorted using BD Influx sorter. Sorted cells were cultured in serum free medium in presence of TPO, SCF in the absence or presence of JQ-1 inhibitor (Clinisciences). At day 2, cells were collected, stained using anti-CD41a-APC and anti-CD42a-PE antibodies (BD Pharmingen) and analyzed using BD Fortessa.

RNAseq samples collection and library preparation

Data generation

For RNAseq, each condition was represented by a randomly selected clone and all the clones were isogenically compared. Cells were labeled with APC, BV421 and PE-conjugated antibodies against CD41a, CD42b and CD33 (see Table S5), respectively. In order to obtain a highly purified megakaryocytes, CD33$^+$ cells were removed from the double positive CD41, CD42 population during cell sorting. After sorting, the purity was verified and corresponded to 98-100% of CD41$^+$, CD42$^+$, CD33$^-$. Total RNA was extracted using RNA/DNA/Protein Purification Plus Kit (Proteigene, St Marcel, France). Whole transcriptome sequencing was performed at the GenomEast Platform (Illkirch, France). cDNA libraries were synthesized from 250 ng total RNA using TruSeq Strandard mRNA Kit (Illumina). Libraries were verified for their amount and quality by capillary electrophoresis using a 2100 Bioanalyzer (Agilent Technologies). Sequencing was performed at 2×100 bp using the Illumina HiSeq 4000 technology, yielding > 40 million reads per sample.

Data analysis

Adapters dimer reads were removed using DimerRemover. Salmon (version 0.14.2) was used for the transcription quantification of the RNA-seq data with an algorithm of selective alignment. To this end, we retrieved the decoy transcriptome (a fasta files associated with a decoy.txt file) generated from the human transcriptome (Grch37/hg19) available at the following link :

https://drive.google.com/drive/folders/14VqSdZAKH82QwDWhMXNLFqMoskoqv3fS

Then, a Salmon index required for the transcription quantification using the command salmon index was generated with the following parameters: -k 31, - -decoys.txt, -t gentrome.fa. Finally, the quantification of reads was performed with the salmon quant command using the following parameters: --seqBias, - -gcBias, -l A –validateMappings.

All subsequent analyses were performed with R (v4.0.3). All subsequent analyses were performed using R (4.0.3). Firstly, an R TxDB object was generated using the function makeTxDbFromGFF of the R package GenomicFeatures (1.42.3). The address used as value of the argument file of this function was: ftp://ftp.ensembl.org/pub/release-97/gtf/homo_sapiens/. The function tximport of the R package tximport (1.18.0) was used to import transcript-level estimates and summarizes abundances, counts, and transcript lengths to the gene-level (with arguments type = "salmon",txOut = FALSE,tx2gene=tx2gene,ignoreTxVersion=TRUE, with tx2gene corresponding to the TxDB object previously generated). An R object of class DESeqDataSet was generated using the function DESeqDataSetFromTximport of the R package DESeq2 (1.30.1). Only genes associated with at least 10 counts were kept (rowSums(counts(dds)) >= 10) for further analysis. Size factors were computed using the function estimateSizeFactors of DESeq2. The vst (variance stabilizing transformation) transformation (implemented in the DESeq2 package) was then applied to the count matrix using the vst function (with argument blind equal to FALSE). A PCA of the vst transformed count matrix was performed using the function PCA (with argument scaling equal to FALSE) of the package FactoMineR (2.4). This matrix was also used for violin plot representations of gene expression as well as input matrix to perform GSEA. Differential expression analysis between experimental conditions was performed with the function DESeq (with default parameters). The function lfcShrink (the value of the parameter type was apeglm) was employed to get estimation of LFC2 (Log2 Fold Change) and of the associated posterior standard deviation (called lfcSE) for each gene between conditions. GSEA was performed using the software GSEA (v4.1.0). Heatmap representations were performed with the package ComplexHeatmap (v2.6.2).

3’ Single-cell RNAseq

Data generation

iPSC-derived CD43-positive cells were sorted at day 13 of culture and prepared at room temperature. Single-cell suspensions were loaded onto a Chromium Single Cell Chip (10x Genomics) according to the manufacturer’s instructions for co-encapsulation with barcoded Gel Beads at a target capture rate of ~10,000 individual cells per sample. Captured mRNAs were barcoded during cDNA synthesis using the Chromium Next GEM Single Cell 3' GEM, Library & Gel Bead Kit v3.1 (10X Genomics) according to the manufacturer’s instructions. All samples were processed simultaneously with the Chromium Controller (10X Genomics) and the resulting libraries were prepared in parallel in a single batch. We pooled all of the libraries for sequencing in a single SP Illumina flow cell. All of the libraries were sequenced with an 8-base index read, a 28-base Read1 containing cell-identifying barcodes and unique molecular identifiers (UMIs), and a 91-base Read2 containing transcript sequences on an Illumina NovaSeq 6000.

Data analysis

1) Counts matrix generation

Adapters sequences were removed using cutadapt (v2.10) with options –nextseq-trim 28 and -a AGATCGGAAGAGCGTCGTGTAGGGAAAGAGTGT

Counts quantification were performed using kallisto (v0.46.2) and bustools (v0.40.0). The index used for alignments was performed thanks to kb-python (v0.40.0) by employing the command kb ref with the fasta and GTF files, respectively available at : ftp://ftp.ensembl.org/pub/release-99/fasta/homo_sapiens/dna/Homo_sapiens.GRCh38.dna. primary_assembly.fa.gz and ftp://ftp.ensembl.org/pub/release-99/gtf/homo_sapiens/ Homo_sapiens.GRCh38.99.gtf.gz.

Bus files were generated using kallisto bus (with option -x 10xv3). They were then sorted using bustools with the command bustools sort (with default behavior that is sort by barcode, UMI, ec then flag). To correct wrong UMIs, the command bustools correct has been used. The file of whitelisted barcodes to correct wrong UMIs was retrieved by downloading cellranger (version 3.1.0 of 10XGenomics). The file 3M-february-2018.txt.gz available with this software was used as the file of whitelisted barcodes. The count matrix was then generated using the command bustools count (with option –genecounts in order to aggregate counts to gene only).

2) Importation, filtration of the matrix and normalization

All subsequent analyses were performed using R (v4.0.3). The function read_count_output of the Bioconductor package BUSpaRse (v1.4.2) was used to generate a sparse matrix

(dgCMatrix) usable for further analyses with R. Droplets associated with counts sum lower than 1000 were removed. Droplets exhibiting less than 200 detected genes were also removed as well as droplets exhibiting more than 20 % of UMIs corresponding to mitochondrial genes. The cell cycle stage associated to each droplet was then predicted with the function CellCycleScoring of the package Seurat (4.0.1) using the lists of genes provided by the function cc.genes.updated.2019 of Seurat. Only genes detected in more than 5 % of cells were kept for further analyses. Doublets detection (identification of droplets containing two cells instead of one) were performed either with the function scDblFinder of the package scDblFinder (v1.4.0) or with the function cxds_bcds_hybrid of the package scds (v1.6.0). Droplets that were tagged as doublets by at least one of these two alternative methods were removed and not used for further analyses. Normalization was performed using the function SCTransform of Seurat (with arguments variable.features.n = 3000, vars.to.regress = NULL and return.only.var.genes = TRUE).

3) Integration of all the single cell datasets (all experimental conditions)

To precisely characterize the differentiation defects according to the different genotypes, an integration of all datasets (one dataset per condition) was performed. To this end, genes used for the integration were selected with the function SelectIntegrationFeatures (Seurat) using all normalized datasets (each one associated to a distinct Seurat object). Seurat objects associated to each dataset where preprocessed using PrepSCTIntegration (Seurat function) before the integration of all datasets. To perform the integration strictly speaking, two functions were used: firstly the Seurat function FindIntegrationAnchor (with argument normalization.method = "SCT") and then the function IntegrateData (with argument normalization.method="SCT"). The function RunPCA (with argument npcs = 20) was used in order to perform a PCA dimensionality reduction of the integration of all datasets. Then the UMAP technique was employed to obtain a projection of the integration (RunUMAP function with arguments reduction = "pca" and dims = 1:20). Classification of cells was performed using the k-nearest neighbors' algorithm with the Seurat function FindNeighbors (with arguments reduction = "pca", dims = 1:20). Then cells were clustered using the SNN algorithm with the Seurat function FindClusters (with argument resolution=1.1).

4) Cell type determination

In order to determine to which kind of cells is associated each cluster of the integration, lists of genes specific to several lineages were used. To determine whether the number of genes detected in these specific lists of genes was higher or lower to what it is expected by chance, a specific method was created. For each dataset used for the integration, 100 randomly selected

lists of genes of the same size as the list of interest were generated (the only criteria to include these genes was that they should be detected in more than 2% of cells of the dataset). For each of these lists, the percentage of detected genes (genes detected at least one time) was computed for each cell. In this way, it led to obtain distributions of the detection rate for random list of genes of the same size as the list of interest. Of note, the mean detection rate of these 100 random lists is expected to be higher for cells exhibiting high sum of counts as compare to cells exhibiting low sum of counts. Therefore, it allows to consider that the detection rate of a specific list of genes does not just depend on the biology of the cell but also of the number of UMIs associated to the cell. For each cell, comparing the detection rate of the list of genes of interest to the distribution of the detection rate of 100 random lists of genes allow to compute a score that determine whether the detection rate observed for a list of genes of interest is significantly higher or lower to what is expected by chance, for a given cell. Applying this method for each cell allows to classify cells (significantly enriched, significantly depleted or neither enriched nor depleted). These classifications were used to identify specific populations of cells in the integrated dataset (for example to identify megakaryocytic cells or erythroid cells).

ATACseq

Data generation

The CD41+/CD42+ MK were sorted (10 000 to 30 000 cells) and submitted to cell lysis, transposition, and purification steps. The transposed DNA fragments were amplified by PCR 12 times using adapters from the Nextera Index Kit (Illumina). PCR purification was performed using MinElute PCR Purification Kit (Qiagen, 28004) to remove large fragments and remaining primers. Library quality was assessed using an Agilent 2100 Bioanalyzer (Agilent Technologies 5067-4626). Libraries were sequenced using NovaSeq-6000 sequencer (Illumina; 50 bp paired-end reads).

Data analysis

ATACseq analysis were performed using the ATACseq nfcore pipeline (nf-core/atacseq: v1.2.1) available at https://nf-co.re/atacseq. Versions of the softwares used by this pipeline are given below: Nextflow (v20.04.1), FastqQC (v0.11.9), Trim Galore ! (v.0.6.4_dev), BWA (v0.7.17-r1188), Samtools (v1.10), BEDTools (v2.29.2), BamTools (v2.5.1), deepTools (v3.4.3), Picard (v2.23.1), R (v3.6.2), Pysam (v0.15.3), MACS2(v2.2.7.1), ataqv (v1.1.1), featureCounts (v2.0.1), Preseq (v2.0.3), Multiqc (v1.9).

Files that has been used by the pipeline are given below :

fasta file: s3://ngi-igenomes/igenomes//Homo_sapiens/UCSC/hg38/Sequence/

WholeGenomeFasta/genome.fa
GTF file: s3://ngi-igenomes/igenomes//Homo_sapiens/UCSC/hg38/Annotation/Genes/genes.gtf
Gene bed file:s3://ngi-igenomes/igenomes//Homo_sapiens/UCSC/hg38/Annotation/Genes/genes.bed
BWA Index: s3://ngi-igenomes/igenomes//Homo_sapiens/UCSC/hg38/Sequence/BWAIndex/genome.fa
Blacklist BED :/home/e_robert@intra.igr.fr/.nextflow/assets/nf-core/atacseq/assets/blacklists/hg38-blacklist.bed
The pipeline was launched using the following command line:
nextflow run nf-core/atacseq --input $pathdesign --genome hg38 --outdir $pathresults -resume -c $pathconfigfile. With this command line, MACS2 (used for the peak calling) performs the peak calling with the mode broad peak.

Results obtained with the ATACseq nfcore pipeline (more specifically MACS2 results) were then used to select differential peaks (associated to an FDR < 0.01) either higher or lower in a condition as compare to another condition (according to the sign of the log2 fold change). For comparisons of interest, peaks were annotated with the command annotatePeaks.pl (Homer v4.11) and motifs analysis was performed employing the command findMotifsGenome.pl (Homer) associated to the option -size 200. The files knownResults.html were used to identify motifs of interest.

Profile plot for scores over sets of genomic regions (list of genes of interest) were performed using the tool plotProfile (deepTools) with the bigwig files generated by the ATACseq nfcore pipeline. Chromosomal locations of list of genes of interest were retrieved using the Bioconductor packages GenomicFeatures (v1.42.3) and TxDb.Hsapiens.UCSC.hg38.knownGene (v3.10.0).

CUT & Tag

Data generation

500 000 $CD41^+$/$CD42^+$ MK sorted cells were used to analyze GATA1 and GATA1s chromatin occupancy using the CUT&Tag-IT Assay Kit (Active Motif) according to the manufacturer recommendations. Briefly, cells were bound to Concanavalin A-Coated Beads and incubated with primary anti-GATA1 antibody in buffer with Protease Inhibitor Cocktail and 5%

digitonine overnight at 4°C under rotation. The Guinea Pig anti-rabbit secondary antibody was incubated in Dig-Wash buffer for 1 hour at RT under rotation. After 3 washes, the CUT&Tag-IT™ Assembled pA-Tn5 Transposomes (1:100) were added for 1 hour at RT under rotation and tagmentation was performed during 1 hour at 37°C. DNA was purified and libraries were generated by PCR. The final libraries were purified, pooled together in equal concentrations and subjected to paired-end sequencing (100 cycles: 2x50) in Novaseq-6000 sequencer (Illumina) at Gustave Roussy.

Data analysis

Fastq files were trimmed using Trim Galore! (v0.6.7). Alignments (hg38 genome) were performed using bowtie2 (2.4.4) with arguments –end-to-end –very-sensitive --no-mixed –no-discordant –phred33 -I 10 -X 700. Sam files were sorted using picard SortSam (Picard v2.25.7). PCR duplicates were removed using picard MarkDuplicates. Sam files were converted into bam files with the command samtools view -S -b 2 (samtools v1.13). To filter unmapped reads and PCR or optical duplicate and keep the mapped read pairs, the command samtools view -b -F 0x04 -F 0x100 was used. Blacklist regions (https://github.com/Boyle-Lab/Blacklist/tree/master/lists/hg38-blacklist.v2.bed) were removed using the command bedtools intersect (bedtools v 2.30.0). Callpeak was performed with MACS2 (v2.2.7.1) using the command macs2 callpeak -t file.bam --qvalue 0.0001 --nomodel --extsize 200 --keep-dup all -f BAMPE.

Supplemental Figures

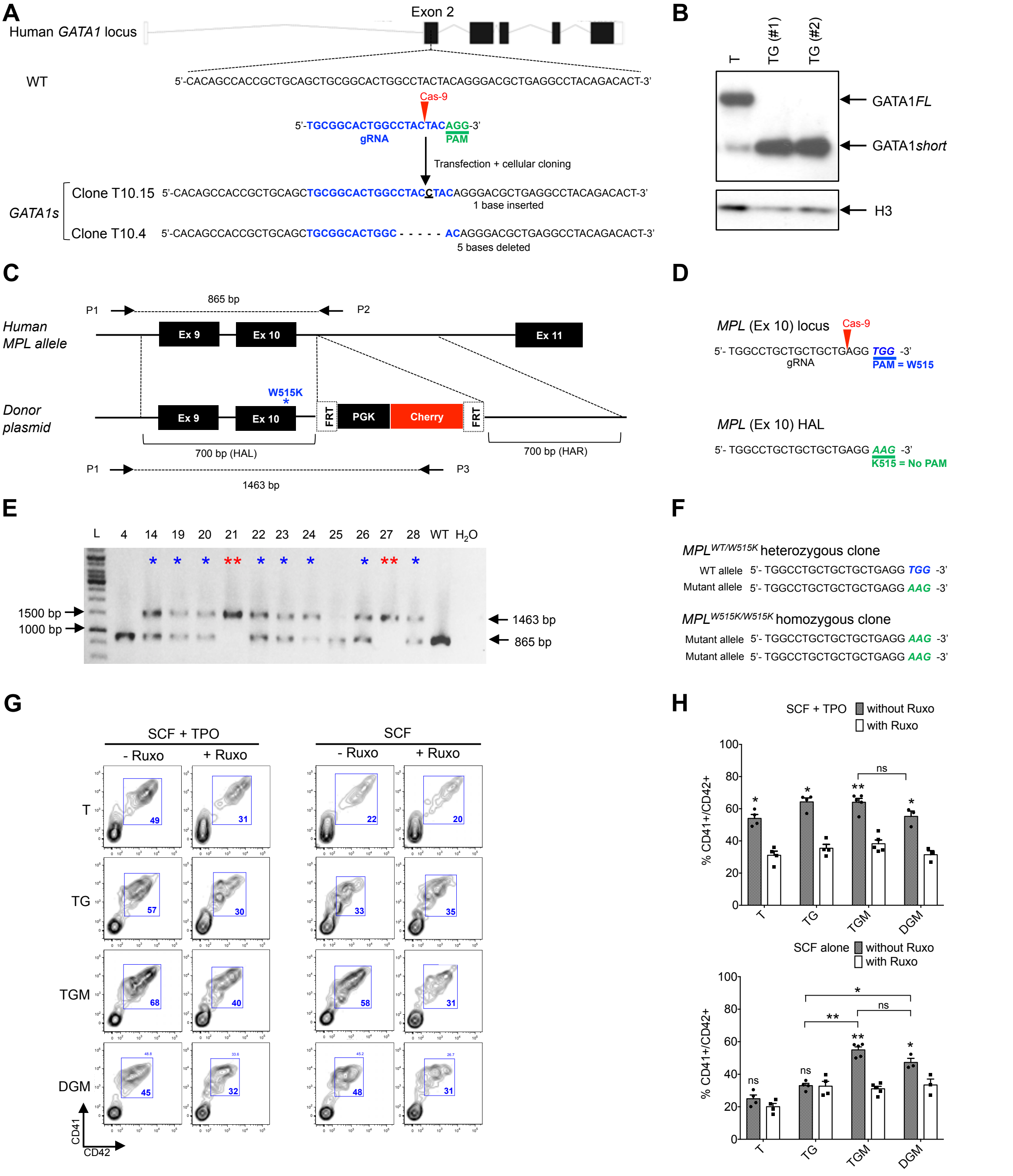

Figure S1. Generation of *GATA1s*, *MPL*^{W515K} and *SMC3*$^{+/-}$ mutations in iPSCs.

(A) Targeting strategy of the exon 2 *GATA1* gene with the designated guide RNA (gRNA) and the resulting Indel sequences. **(B)** Immunoblots for GATA1 protein isoforms in T and T *GATA1s* iPSC clone-derived MK (iMK). Histone 3 (H3) was used as an endogenous control. **(C)** The Knock-In strategy used for the generation of *MPL*$^{W515K/W515K}$ mutation in one of the T21 *GATA1s* iPSC clones. The primers P1, P2 and P3 were used for the genotyping of Cherry-positive selected clones: Wild type allele amplified with P1 and P2 (865 bp); Mutant allele amplified with P1 and P3 (1463 bp).**(D)** The Knock-In associated-guide RNA (gRNA) targeting exon 10 of *MPL* gene. The PAM corresponded to the W515 (TGG), which was replaced by K515 (AAG) present in HAL of the donor plasmid. **(E)** Identification of the Cherry-positive clones after PCR electrophoresis. The blue stars show heterozygous *MPL*$^{WT/W515K}$ clones (bands: 865 bp + 1463 bp) while the red stars show homozygous *MPL*$^{W515K/W515K}$ clones (band: 1463 bp). A wild type clone control was integrated and shows only the band at 865 bp. **(F)** Example of the *MPL*$^{WT/W515K}$ or *MPL*$^{W515K/W515K}$ mutant clones obtained after Sanger sequencing. **(G)** Representative flow cytometry analysis of MK cultures obtained in the presence of SCF alone or SCF+TPO +/- Ruxolitinib (Ruxo). **(H)** Histograms of the percentage of CD41^{+}CD42^{+} obtained in the indicated conditions. Results are represented as mean ± SEM with n=5.

Figure S1 (continued)

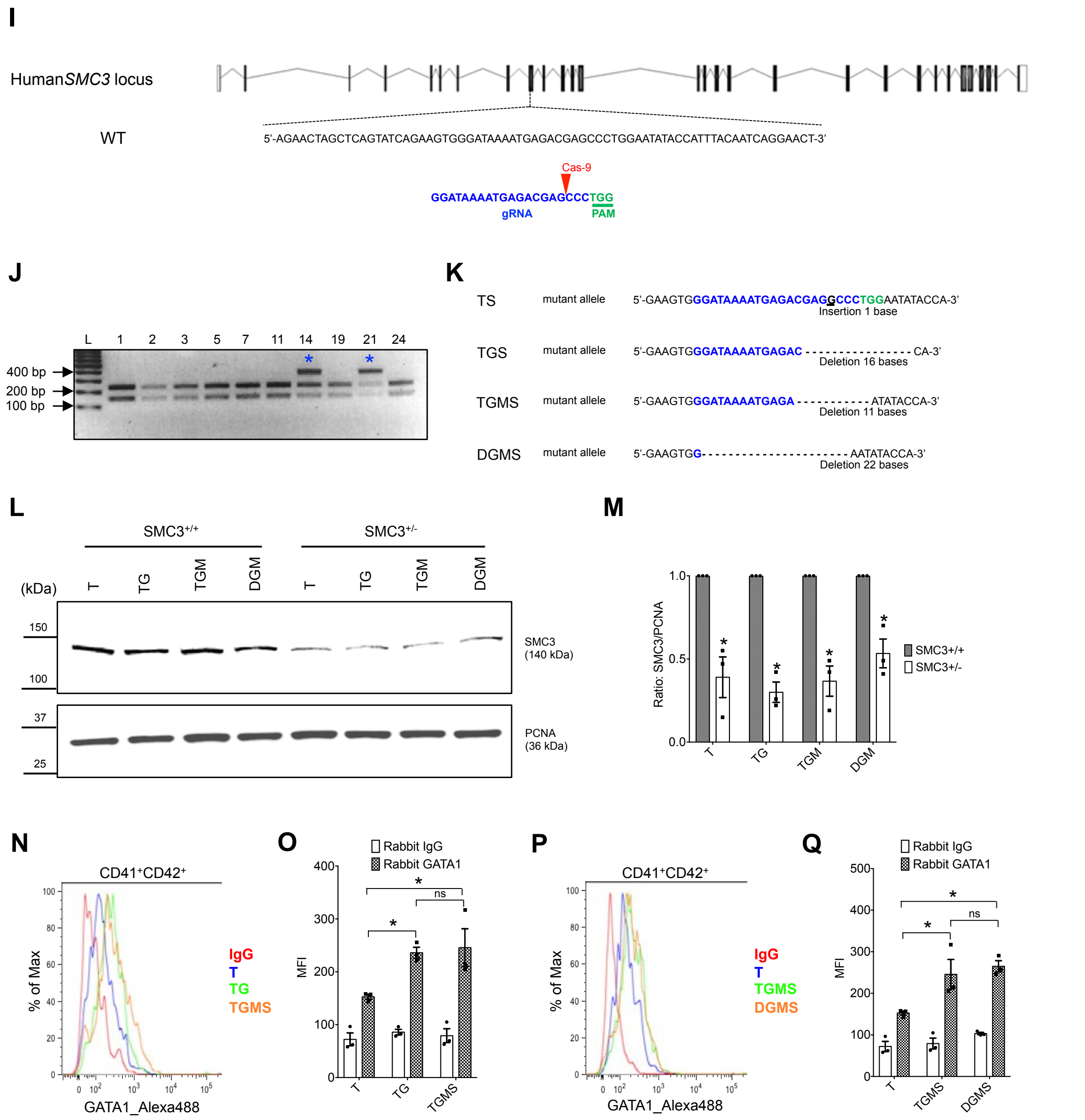

(I) Targeting strategy of exon 9 *SMC3* gene with the designated gRNA. **(J)** Genotype analyses of GFP-positive transfected iPSC clones using BanII restriction enzyme. The stars show heterozygous *SMC3* mutant clones (#S14, #S21) containing either the non-digested (BanII restriction site modified by Cas-9 endonuclease; presence of indel) or digested allele (BanII restriction site remained WT) at 381 bp or 236 bp and 145 bp, respectively. **(K)** Example of the resulting Indel sequences per genotype containing $SMC3^{+/-}$ mutation. **(L)** Immunoblot for SMC3 in $SMC3^{WT}$ and $SMC3^{+/-}$ iPSC clones. PCNA was used as an endogenous control. **(M)** Semi-quantitative evaluation of results shown in E. For each genotype, the SMC3 band intensity was normalized using PCNA band intensity and is represented relative to the $SMC3^{+/+}$ condition. The data are represented as the mean ± SEM (*n* = 3). **(N)** Intracellular flow cytometry analyses of $GATA1^{WT}$/GATA1s protein expression in T, TG and TGMS $CD41^+CD42^+$ MK population. **(O)** Histogram shows the mean fluorescence intensity of $GATA1^{WT}$/GATA1s in the indicated conditions. The data are represented as the mean ± SEM (*n* = 3). **(P)** Flow cytometry analyses of $GATA1^{WT}$/GATA1s protein expression in T, TGMS and DGMS $CD41^+CD42^+$ MK population. **(Q)** Histogram shows the mean fluorescence intensity of $GATA1^{WT}$/GATA1s in the indicated conditions. The data are represented as the mean ± SEM (*n* = 3).

Figure S1 (continued)

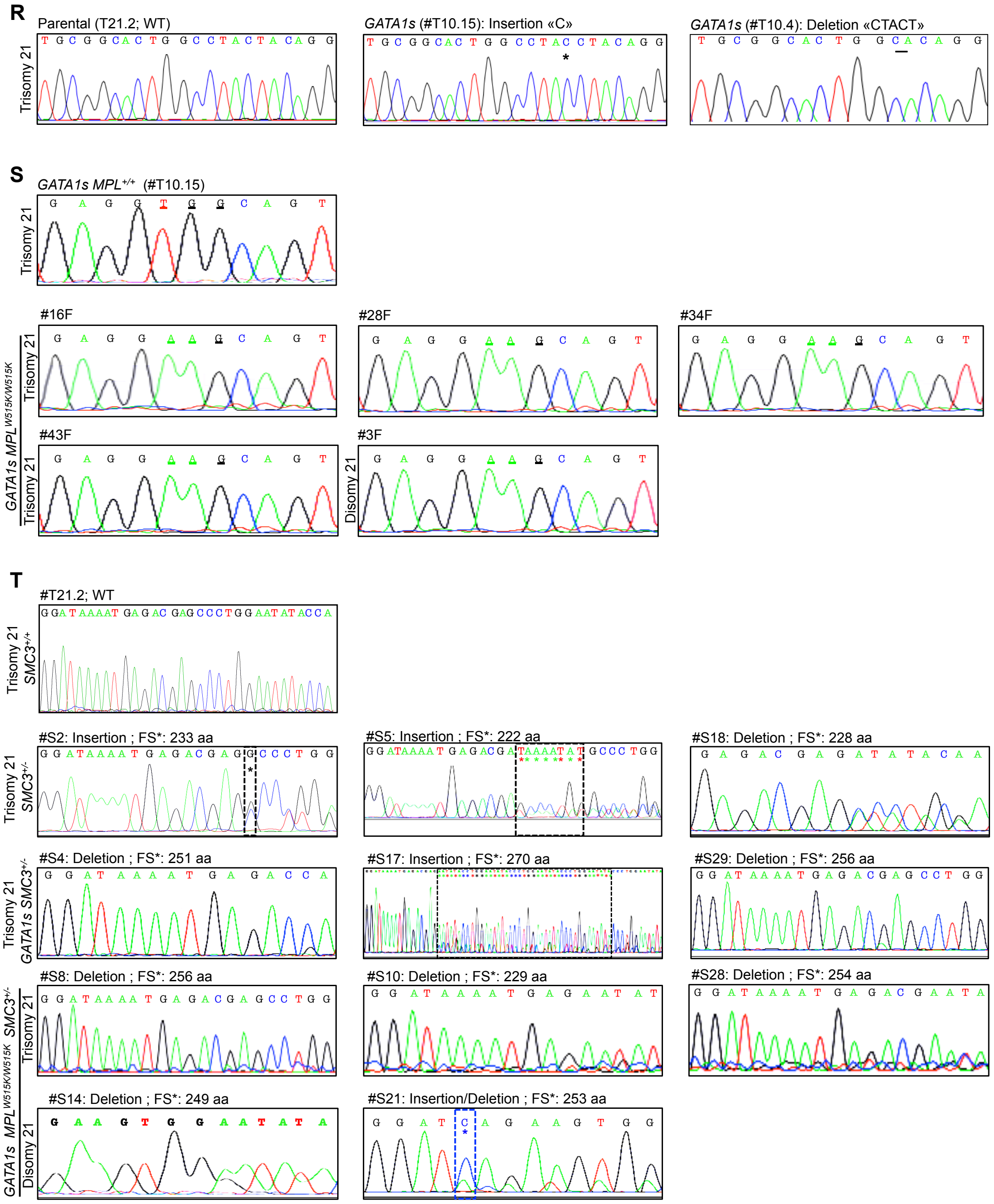

(R) Sanger sequencing results showing the indels obtained for TG clones. The sequence of T (parental) iPSC clone is also shown as a control. **(S)** Sanger sequencing results showing the *MPL*$^{W515K/W515K}$ mutant clones in the T or D contexts. The substitution of “TGG” (W515) by “AAG” (K515) in exon 10 of the *MPL* gene is shown for each clone. The corresponding wild type sequence (*MPL*$^{WT/WT}$) of TG iPSC clone is also shown as a control. **(T)** Sanger sequencing results showing the *SMC3*$^{+/-}$ indels obtained for T, TG, TGM and DGM clones. The corresponding wild type sequence (*SMC3*$^{+/+}$) of T iPSC clone is also shown as a control.

Figure S1 (continued)

U

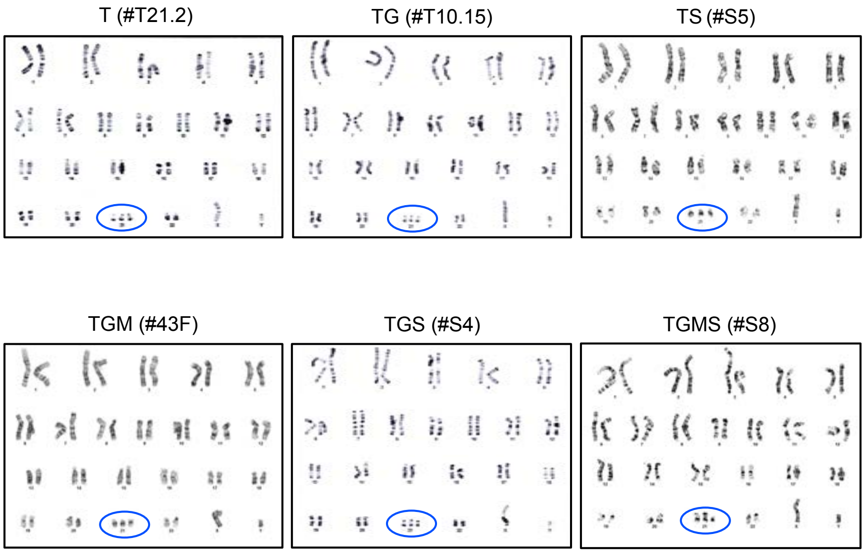

V

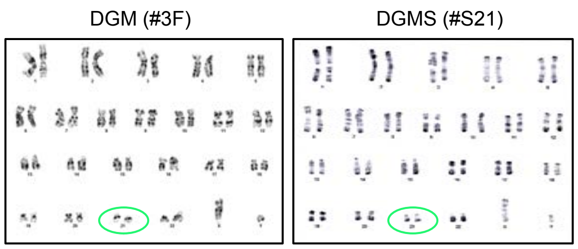

W

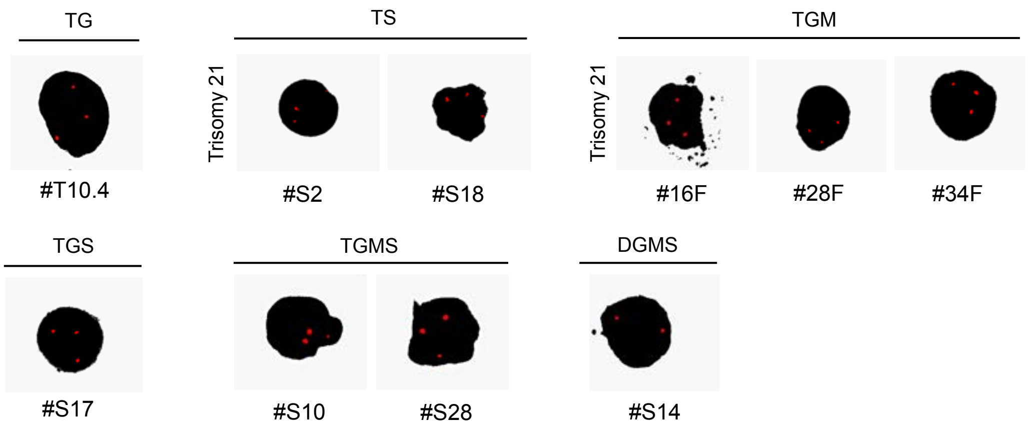

(U) Karyotypic analyses of T (parental), TS, TG, TGM and TGMS iPSC clones. **(V)** Karyotypic analyses of DGM and the subsequent DGMS iPSC clones. **(W)** Fluorescent *In Situ* Hybridization (FISH) analyses for the presence or absence of the trisomy 21. Statistical significance was determined using one-tailed Mann-Whitney's U test: $p<0.05$ (*), $p<0.01$ (**), $p<0.001$ (***).

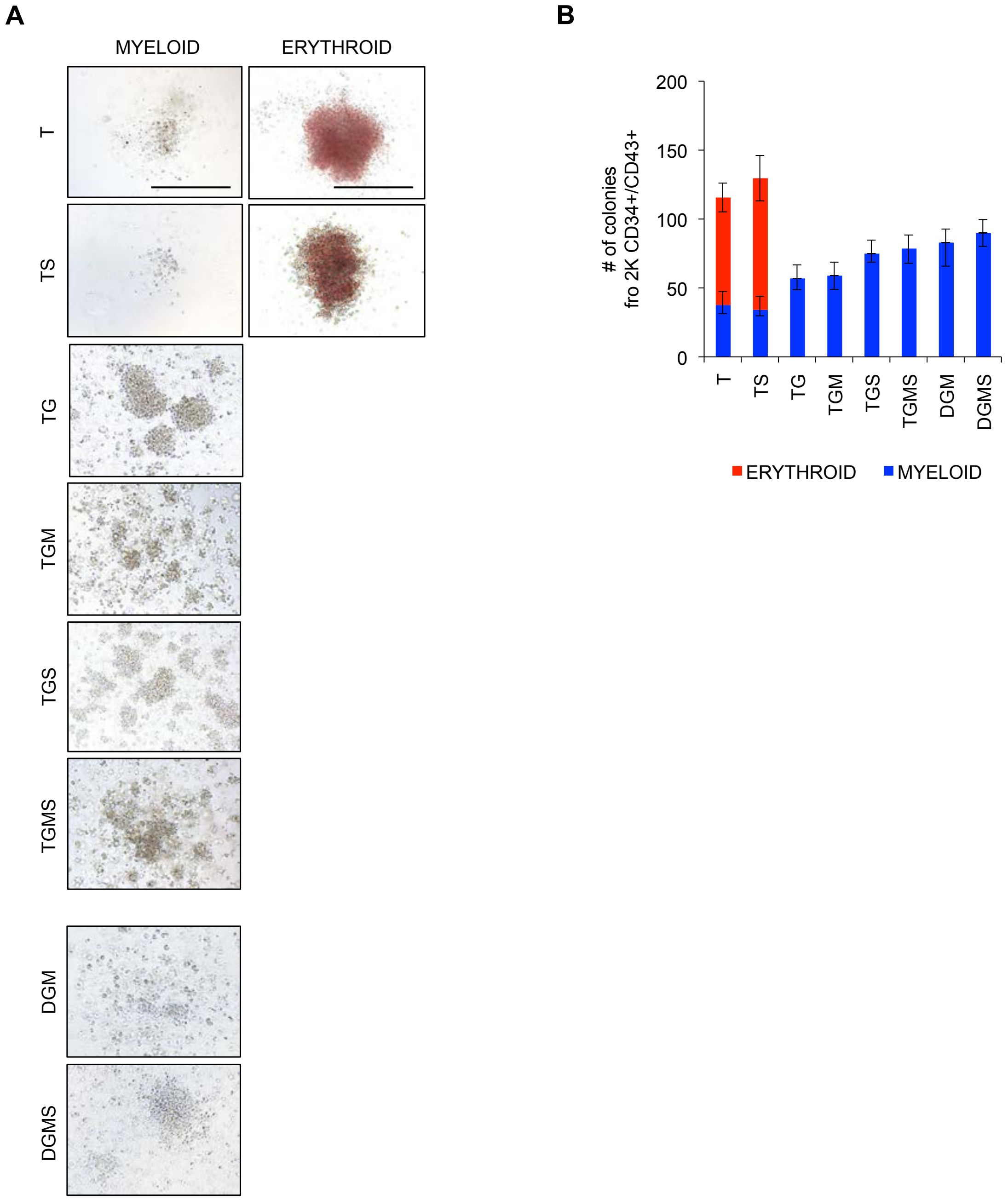

Figure S2. Clonogenic potential of iPSC-derived hematopoietic progenitors according to the different genotypes.

(A) Representative microphotographs of CFU-Myeloid (left panel) and Erythroid (right panel) colonies among the different genotypes. Scale bars represent 500 µm. **(B)** Histogram showing the number of Erythroid and Myeloid colonies from 2000 $CD34^{+}CD43^{+}$, in methylcellulose culture assays.

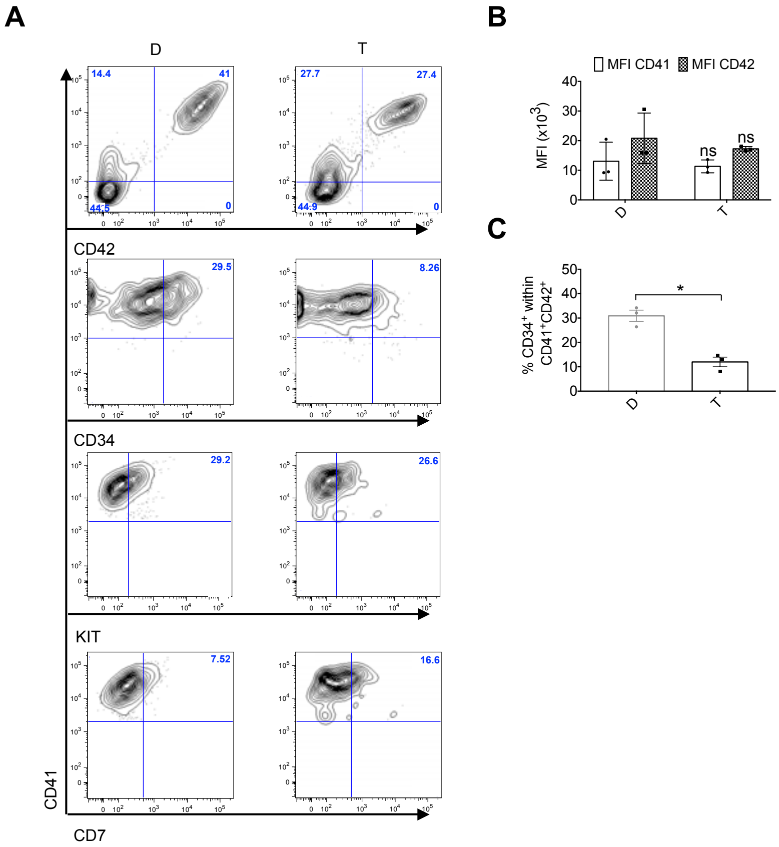

Figure S3. Assessment of iMK differentiation

(A-C) Immunophenotypes of iMK for the CD34, KIT, CD7, CD41 and CD42 markers found in DS-AMKL blasts. **(A)** Contour plots showing the expression of CD34, CD41, CD42, KIT and CD7 markers. **(B)** Histogram shows the MFI of CD41 and CD42. **(C)** Histogram shows the percentage of $CD34^+CD41^+$ per total $CD41^+$ population. The results in B and C are represented as the mean ± SEM, n = 3. Statistical significance was determined using one-tailed Mann-Whitney's U test: p<0.05 (*), p<0.01.

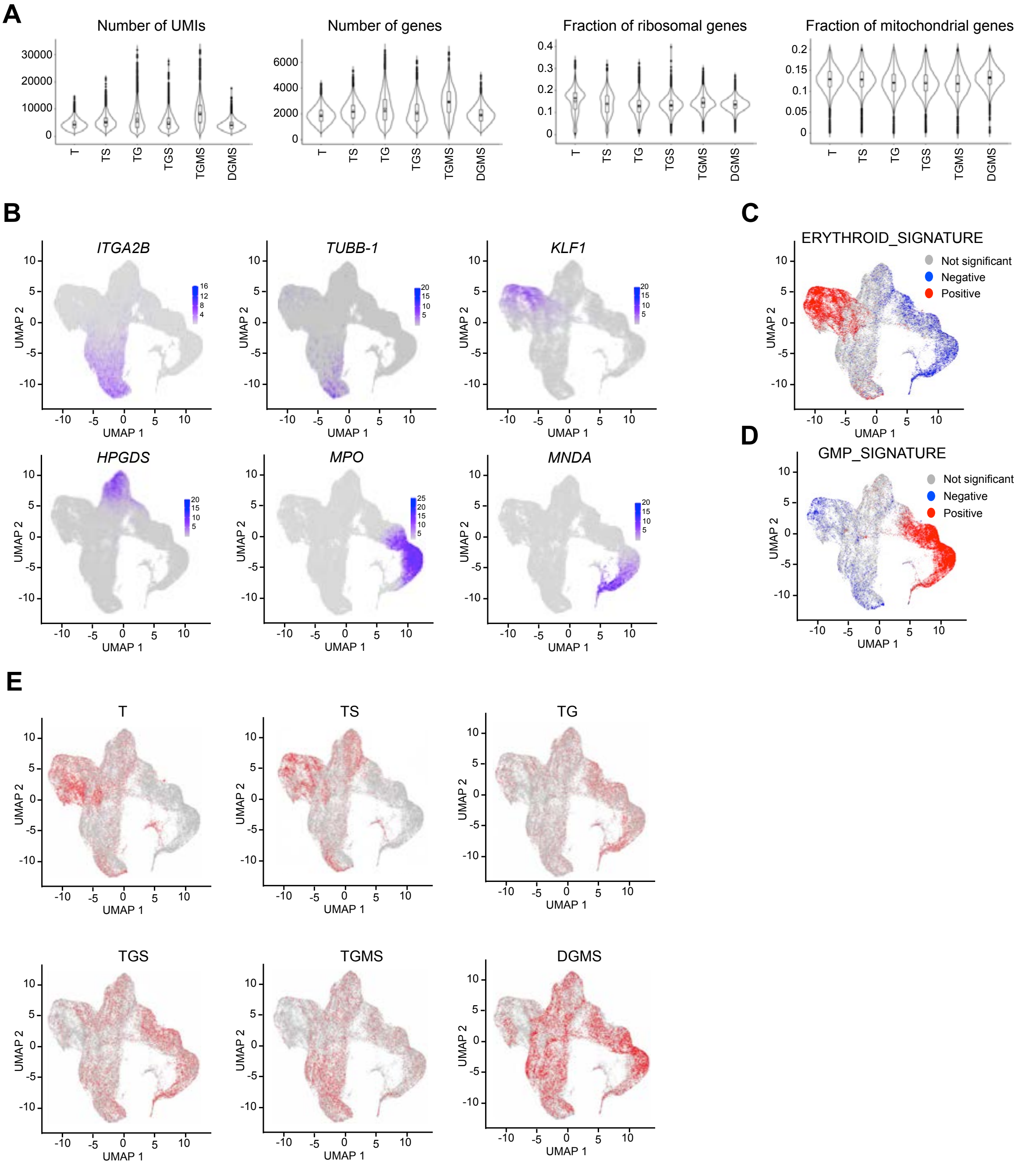

Figure S4. scRNAseq of the iPSC-derived CD43^{+} hematopoietic cells on day 13 of culture.

(A) Violin plots showing either the number of UMIs or genes and the fractions of ribosomal or mitochondrial genes in the indicated conditions. **(B)** UMAP of the integration: points are colored according to the expression level of the indicated genes. **(C-D)** UMAP of the integration: the points colored in red are significantly enriched either for erythroid **(C)** or GMP **(D)** signatures. Points in blue are significantly depleted for the respective lineage signatures. Points in grey are not significant. **(E)** UMAP of the integration showing the localization (in red) of the cells from each data set in the integration.

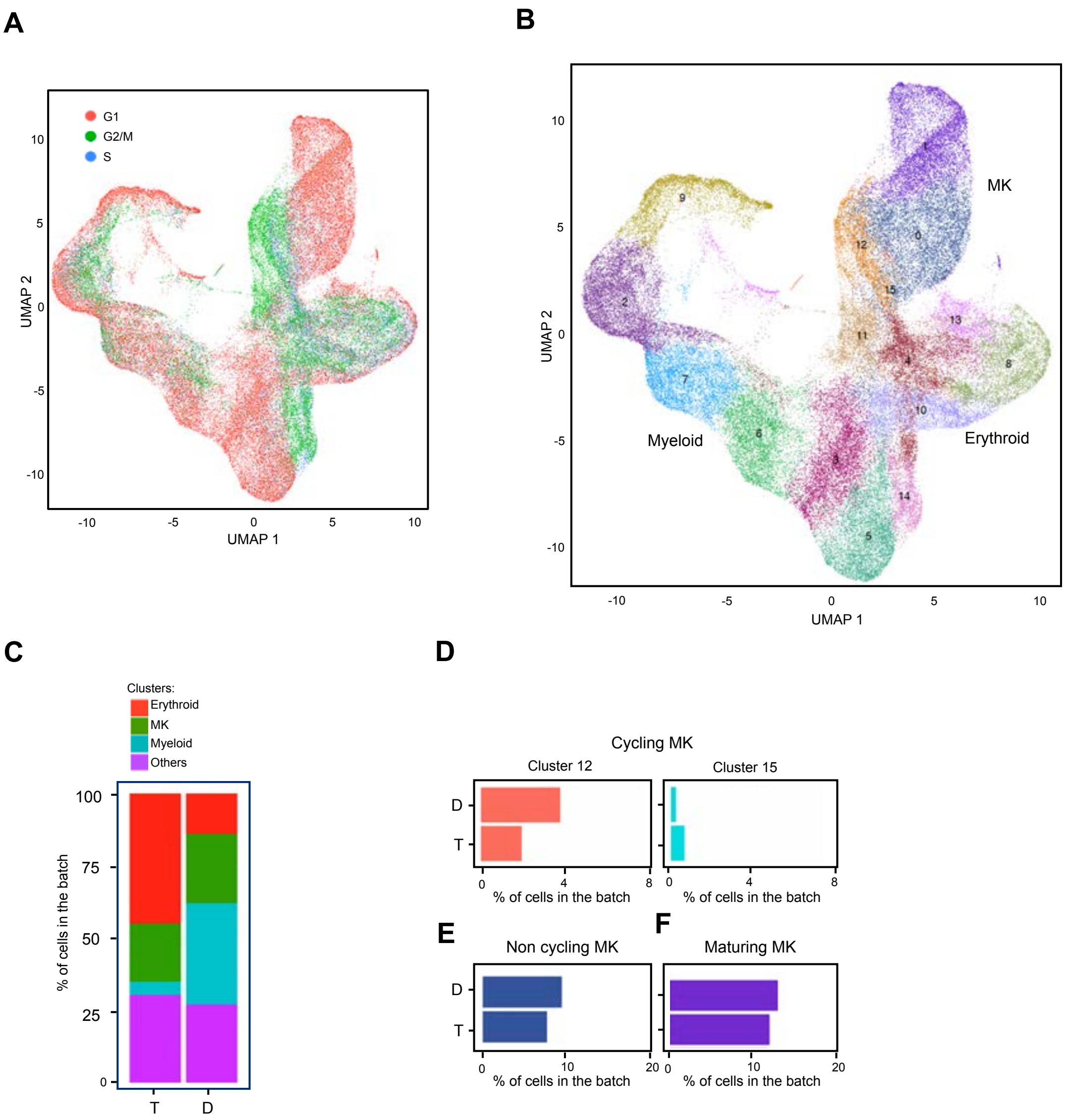

Figure S5. scRNAseq of CD43⁺ iPSC-derived hematopoietic cells at day 13 of culture.
(A) UMAP integration with cells colored according to the predicted cell cycle stage (Seurat method). **(B)** UMAP integration of cells from D and T. Clusters were defined using the Louvain algorithm, numbered and labelled with unique colors. **(C)** Bar plot shows the proportion of cells in the indicated hematopoietic lineages for D and T. **(D)** Bar plots of the proportion of cells in the two clusters of cycling MK. **(E)** Bar plots of the proportion of cells in the cluster of non-cycling MK. **(F)** Bar plots of the proportion of cells in the cluster of maturing MK.

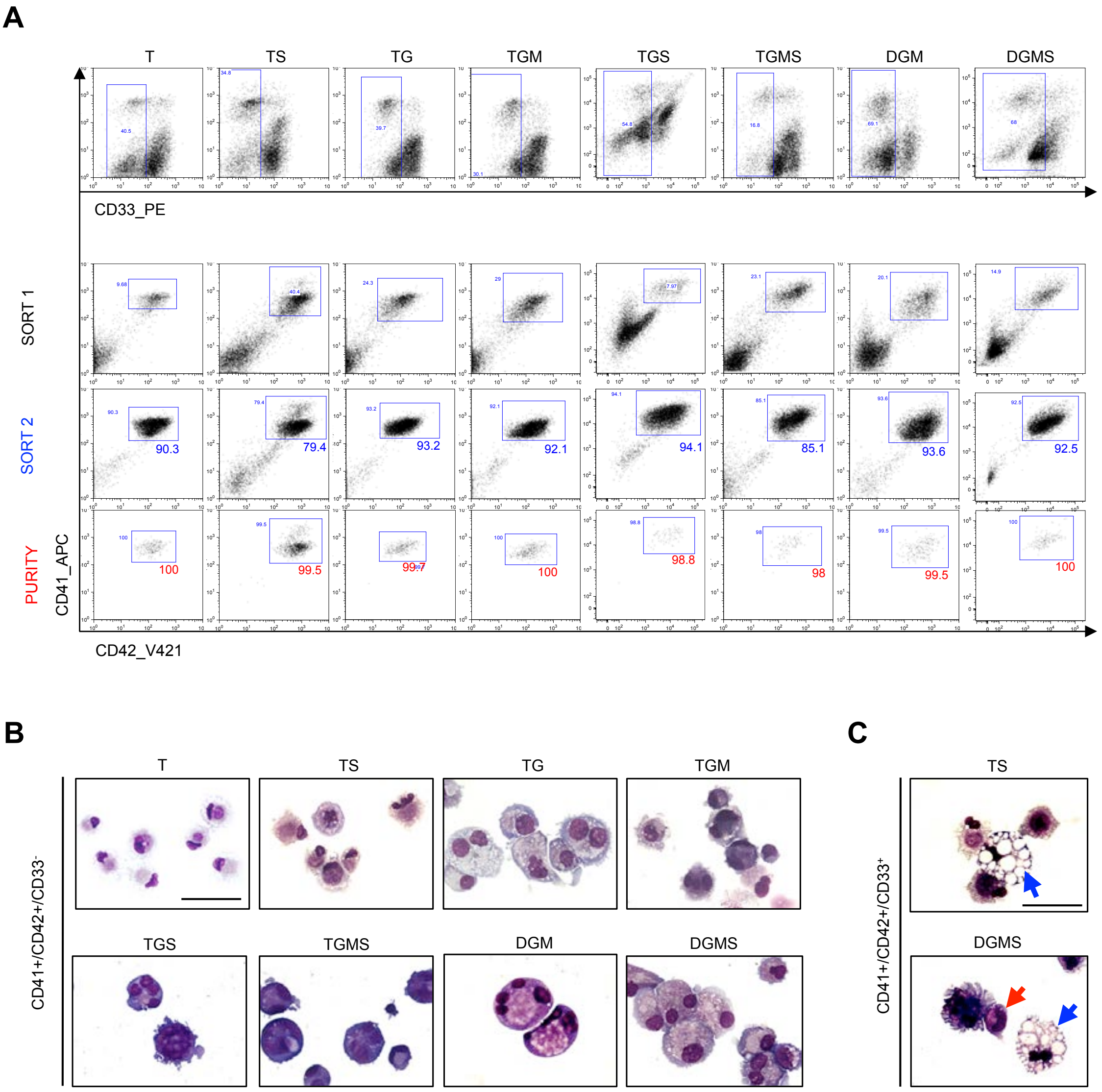

Figure S6. RNAseq analyses of iPSC-derived MK according to the different genotypes.

(A) Gating and sorting strategies used to obtain a pure MK population. CD41$^+$CD33$^+$ were excluded from the sorting; keeping only the CD41$^+$CD42$^+$CD33$^-$ cells. At the end of the first cell sorting, the purity varied from 79% to 94%, while a homogenous purity of nearly 100% was reached for all the genotypes at the end of the second cell sorting. **(B)** May Grünwald Giemsa coloration of the cytospinned CD33$^-$ fraction from CD41$^+$CD42$^+$ MK population. Note the presence of MK without any contaminating cells. Scale bar: 50 μm. **(C)** May Grünwald Giemsa coloration of the cytospinned CD33$^+$ fraction from CD41$^+$CD42$^+$ MK population. Note the presence of macrophage (blue arrowhead) or granulocyte (red arrowhead) contaminating cells. Scale bar: 50 μm.

Figure S6 (continued)

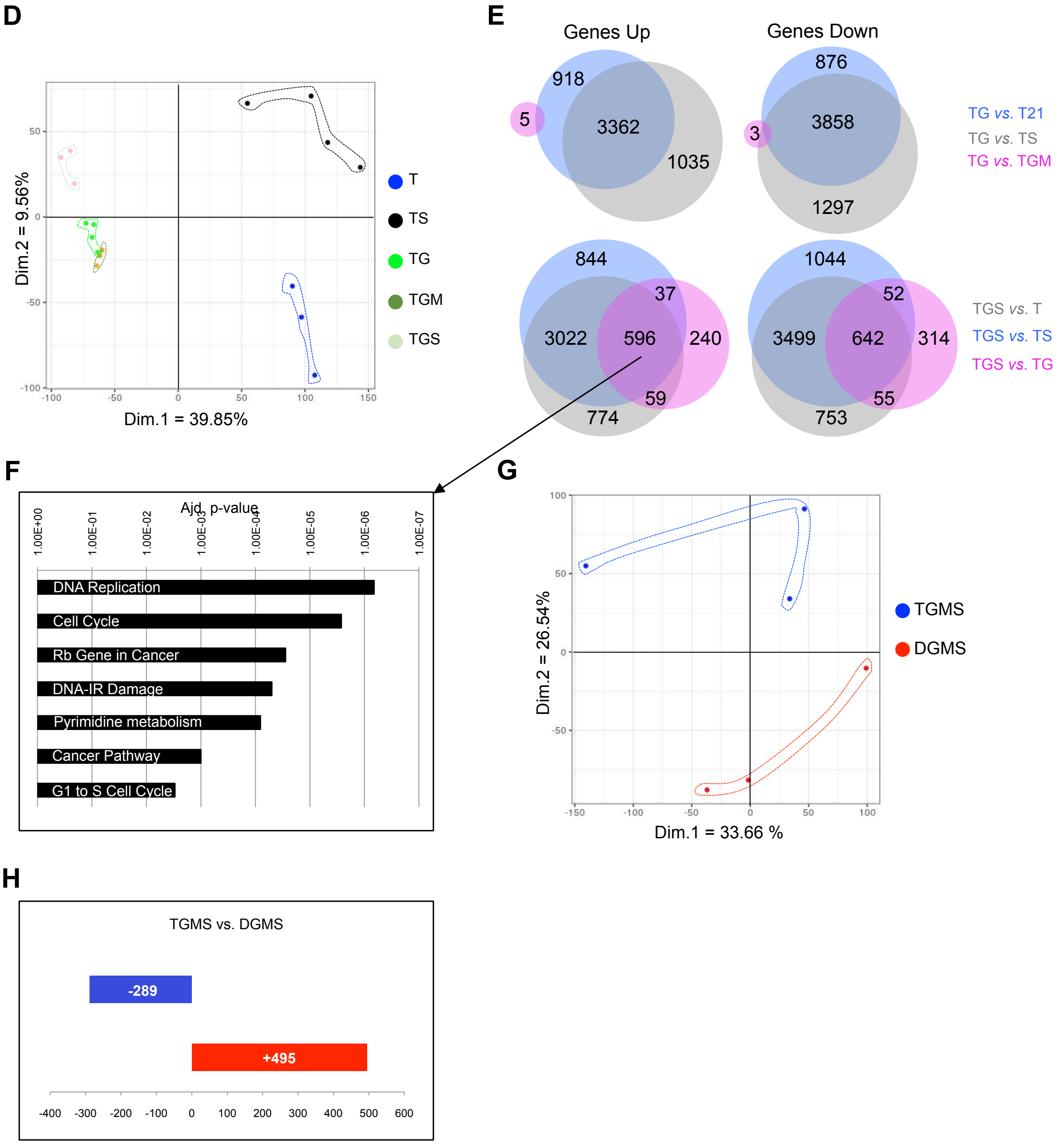

(D) Principal Component Analyses (PCA) of RNAseq data obtained from T, TS, TG, TGM and TGS-derived MK. RNAseq was performed on three to four biological replicates for each condition. **(E)** Venn diagram illustrates common and differentially expressed genes. Significantly up/ downregulated genes (adjusted *p*-value < 0.05) were compared between the indicated conditions. **(F)** Transcriptional signatures obtained from the commonly upregulated genes in TGS compared either to T, TS or TG. **(G)** PCA of RNAseq data obtained from TGMS and DGMS-derived MK. RNAseq was performed on three biological replicates for each condition. **(H)** Bar plot showing the number of up- or downregulated genes in TGMS compared to DGMS-derived MK. Adjusted *p*-value < 0.05.

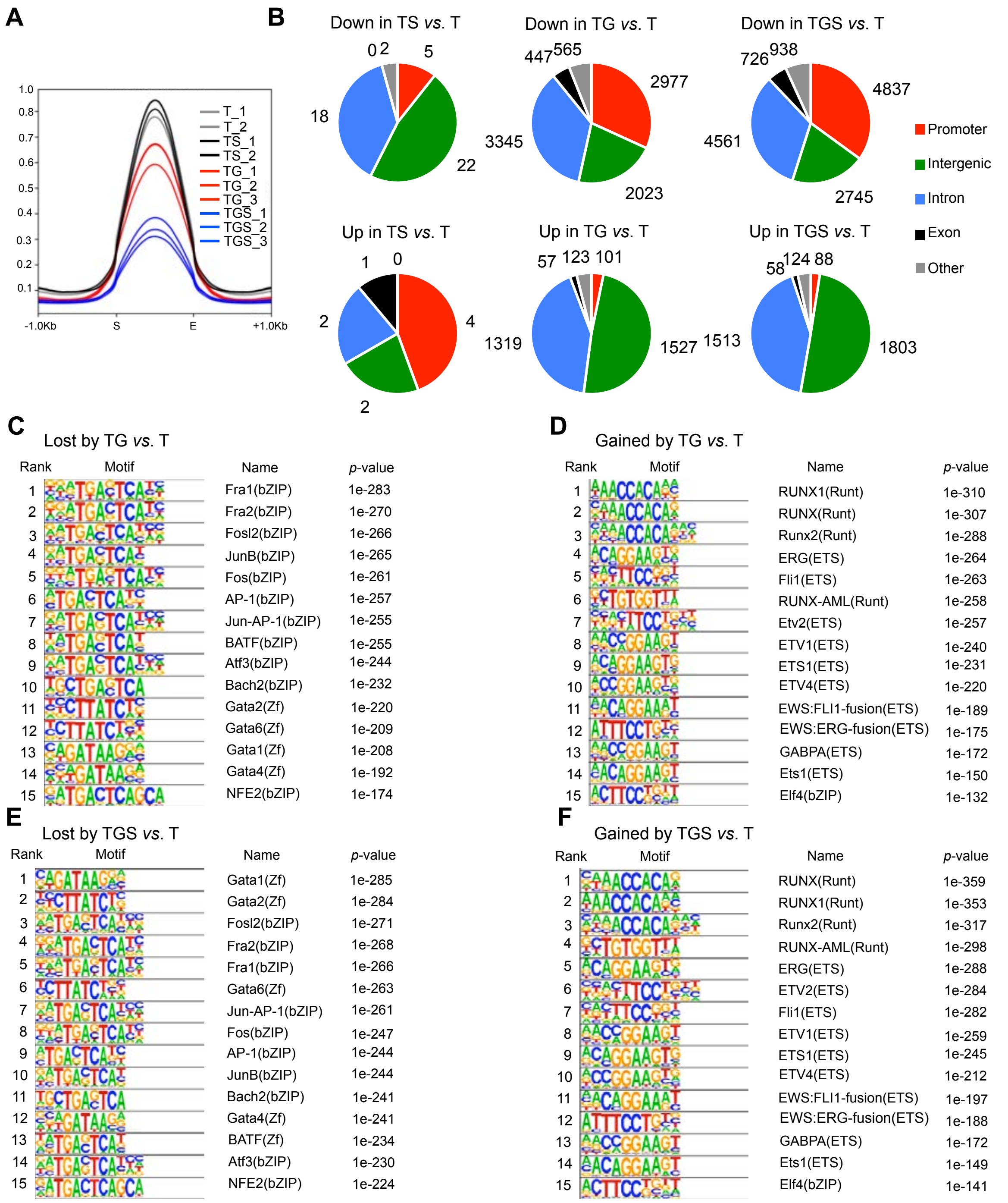

Figure S7: ATACseq analyses of iPSC-derived MK.

(A) Metaplot of ATAC peak signal in T compared to either TS, TG or TGS iPSC-derived MK. The start (S) and the end (E) of the narrow peaks were plotted across a 1-Kb flanking window; the y-axis indicates depth per million mapped reads. Two replicates are shown for T and TS, and three replicates are shown for TG and TGS. **(B)** Pie charts showing the distribution of accessible sites that were gained or lost in TS *vs*. T, TG *vs*. T and TGS *vs*. T. **(C)** Rank list of motifs that lost accessibility in TG *vs*. T. **(D)** Rank list of motifs that gained accessibility in TG *vs*. T.**(E)** Rank list of motifs that lost accessibility in TGS *vs*. T. **(F)** Rank list of motifs that gained accessibility in TGS *vs*. T.

Figure S7 (continued)

G

Lost by TGS *vs.* TG

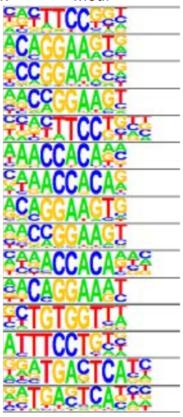

Rank	Name	*p*-value
1	Fli1(ETS)	1e-139
2	ERG(ETS)	1e-136
3	ETV4(ETS)	1e-124
4	ETV1(ETS)	1e-119
5	ETV2(ETS)	1e-117
6	RUNX1(Runt)	1e-114
7	RUNX(Runt)	1e-108
8	ETS1(ETS)	1e-107
9	GABPA(ETS)	1e-96
10	RUNX2(Runt)	1e-90
11	EWS:FLI1(ETS)	1e-89
12	RUNX-AML(Runt)	1e-80
13	EWS:ERG(ETS)	1e-76
14	Fra2(bZIP)	1e-72
15	Fosl2(bZIP)	1e-69

H

Up by TGS *vs.* TG

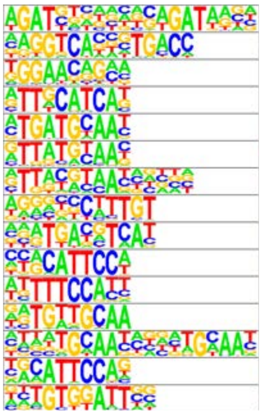

Rank	Name	*p*-value
1	GATA3(Zf)	1e-20
2	ERE(NR)	1e-9
3	ZNF189(Zf)	1e-9
4	Chop(bZIP)	1e-7
5	Atf4(bZIP)	1e-7
6	HLF(bZIP)	1e-5
7	NFIL3(bZIP)	1e-5
8	Sox9(HMG)	1e-5
9	CREB5(bZIP)	1e-4
10	TEAD1(TEAD)	1e-3
11	NFAT(RHD)	1e-3
12	CEBP:AP1(bZIP)	1e-3
13	CEBP(CEBP)	1e-3
14	TEAD3(TEA)	1e-3
15	Foxh1(Forkhead)	1e-3

(**G**) Rank list of motifs that lost accessibility in TGS *vs*. TG. (**H**) Rank list of motifs that gained accessibility in TGS *vs*. TG.

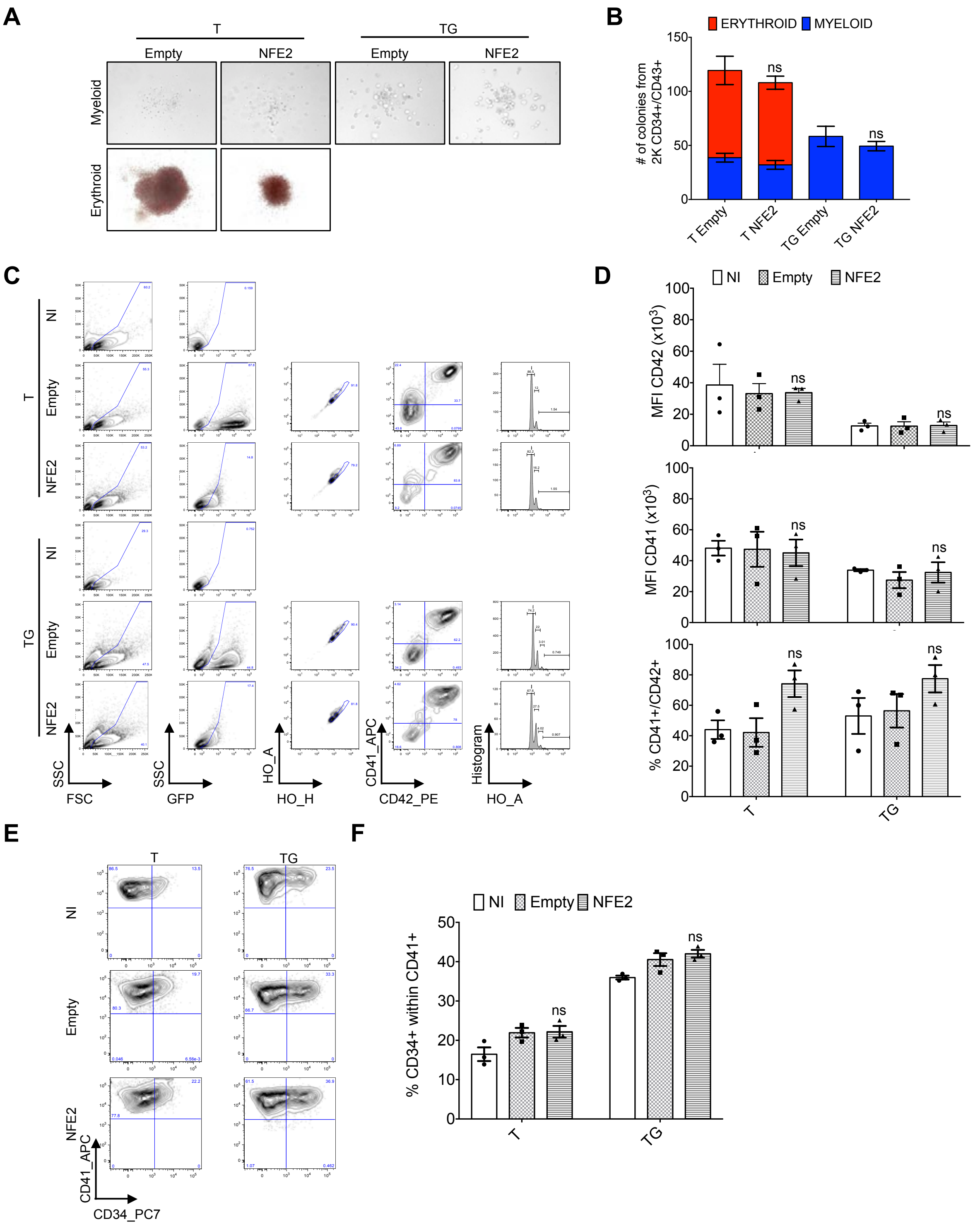

Figure S8. Effects of NFE2 overexpression on T or TG-derived MK.

(A) Photomicrographs showing myeloid or erythroid colonies from T or TG transduced either with the empty or the NFE2 lentiviral vector. Scale bar: 500 μm. **(B)** Histogram showing the mean number of CFU-erythroid or myeloid colonies from T or TG transduced either with the empty or the NFE2 lentiviral vector. The results are represented as the mean ± SEM, n = 3. ns: not statistically significant. **(C)** Gating strategy used to assess MK ploidy in the conditions tested. NI: No infection. **(D)** Mean percentage and Mean Fluorescence Intensity (MFI) of CD41/CD42 according to the compared conditions. The results are represented as the mean ± SEM, n = 3. ns: not statistically significant, NI: No infection. **(E)** Contour plots showing the CD34$^+$ cell fraction from total CD41$^+$ MK. NI: No infection. **(F)** Histogram shows the mean percentage of CD34$^+$/CD41$^+$ from the total CD41$^+$ MK. The results are represented as the mean ± SEM, n = 3. ns: not statistically significant, NI: No infection.

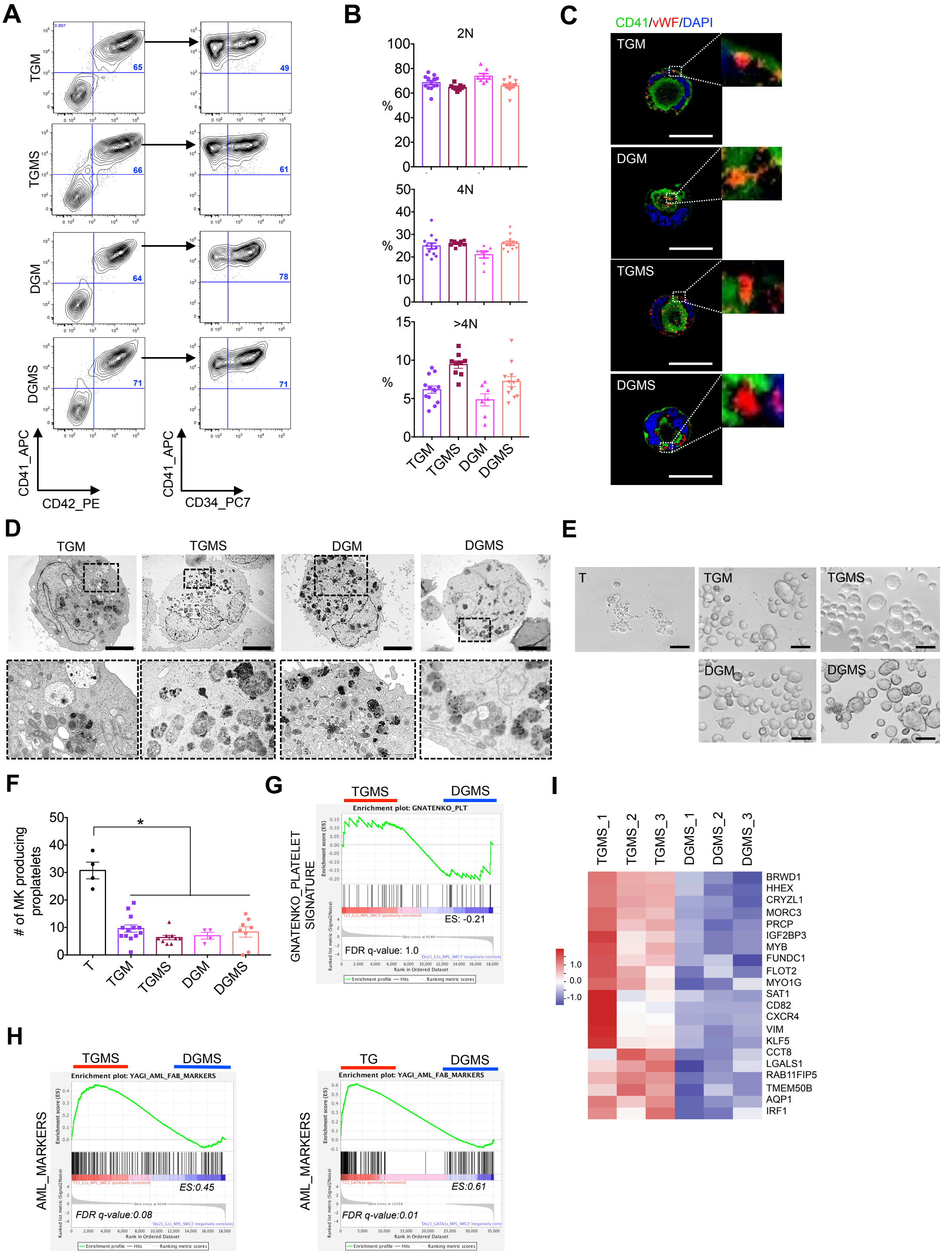

Figure S9. Role of trisomy 21.

(A) Contour plots showing the percentage of $CD34^+$ fraction from $CD41^+CD42^+$ MK according to the indicated genotypes. **(B)** Histogram shows the mean percentage of 2N, 4N and >4N MK in the indicated genotypes. **(C)** Confocal analyses of CD41 and vWF expression in MK according to the indicated genotypes. Scale bars: 50 µm. **(D)** Ultrastructural characterization of iPSC-derived MK in the indicated genotypes. One MK is shown for each condition (**upper panel**) and a part of which (dotted square) is enlarged (**lower panel**). Note the presence of the typical TG MK maturation defects (described in figure 3E) in TGM, TGMS, DGM and DGMS conditions. Scale bars: 5 µm. **(E)** Representative microphotographs of $CD41^+CD42^+$ MK at day 3 of culture. Scale bars: 50 µm. **(F)** Histogram shows the number of MK forming proplatelets according to the different genotypes. The results are represented as the mean ± SEM, n = 3 to 4. The number of clones tested per genotype is: 4 (TGM), 3 (TGMS), 1 (DGM), 2 (DGMS). **(G)** GSEA of platelet marker genes (Gnatenko et al., 2003) in TGMS *vs*. DGMS-derived MK. **(H)** GSEA of AML gene markers in the indicated comparisons. **(I)** Heatmap shows the top 21 gene marker of AML in TGMS *vs*. DGMS-derived MK.

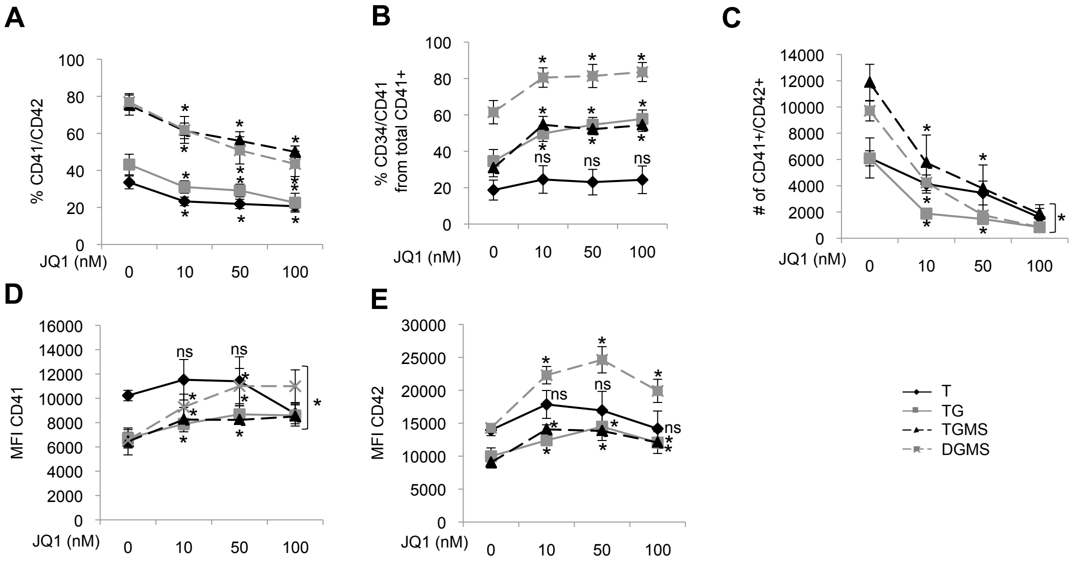

Figure S10. Assessment of JQ-1 concentrations on MK outcome

(A) Curve graph shows the percentage of CD41$^+$ CD42$^+$ according to the different concentrations of JQ-1 inhibitor. **(B)** Percentage of CD34$^+$ within the CD41$^+$ CD42$^+$ population according to the different concentrations of JQ-1 inhibitor. **(C)** Absolute number of CD41$^+$ CD42$^+$ according to the different concentrations of JQ-1 inhibitor. **(D)** Mean fluorescence intensity of CD41 according to the different concentrations of JQ-1. **(F)** Mean fluorescence intensity of CD42 according to the different concentrations of JQ-1. The results are represented as the mean ± SEM, $n = 3$. Each JQ-1 concentration ranging from 10 to 100 nM was statistically compared to 0 nM in each condition Statistical significance was determined using one-tailed Mann-Whitney's U test: $p<0.05$ (*).

Supplemental Tables

Table S1: List of off-target sites verified for all the CRISPR-edited iPS clones (related to Figure S1).

Targeted gene	iPSC lines tested for off-targets	Off-target site	Off-target sequence	Positions of the mismatches on the off-target sequence	Result: Sanger sequencing
GATA1	T21 GATA1s p. Y62Qfs*4 and p. Y63Lfs*5	3: intron: ASAP1	*CTGCAACACTGCCCT ACTAC AGG*	____**_____*________	Wild type
		4: exon: SREBF2	ATGCGGCTCTGGCC TATCAC *CGG*	*_______*_______**__	Wild type
		4: exon: XPNPEP2	ATGCTGCCCTGGCC CACTAC *AGG*	*___*__*______*_____	Wild type
		4: intron: DNAH8	CTGTGTCAGTGACC TACTAC *TGG*	___*_*__*__*________	Wild type
		4: exon: THOC5	CTCCGGCAGTGGCT TACTCC *CGG*	__*_____*____*____*_	Wild type
MPL	T21 and Dis21 GATA1s p. Y63Lfs*5 MPL p. W515K	3: exon: BRAP	AGGAGCTGCTGCTG CTGAGG *CGG*	*__**_______________	Wild type
		3: exon: GABRG1	TCAGCCAGCTGCTG CTGAGG *TGG*	_**___*_____________	Wild type
		2: exon: TTC34	TGGCCCTGCTGCAG CTGAGG *AGG*	___*________*_______	Wild type
		3: exon: BACH2	TGCTGCTGCTGCTG CTGAGG *CGG*	__***_______________	Wild type
		3: intergenic:AC138647.1-AC104417.1	CGGGCTTCCTGCTG CTGAGG GGG	*____*_*____________	Wild type
		2: intergenic:AC106860.1-RP11-234O6.2	TGGGTCTGCTGCTG CTGAGC TGG	___*_______________*	Wild type
		3: intron:DVL1	TGCAGCTGCTGCTG CTGAGG AGG	__***_______________	Wild type
		3: intron:SDK2	TGGCTCAGCTGCTG CTGAGG AGG	___**_*_____________	Wild type
		3: intergenic:RP11-719N22.2_SRP	TAGGTCTGCTTCTG CTGAGG AGG	___**_*_____________	Wild type
		3: intergenic:TBC1D22A-RP1-111J24.1	TGGGGCTGCAGCTG CTCAGG TGG	____*____*______*___	Wild type
		2: intergenic:NSDHL-ZNF185	TGGGGCTGCTGCTG CTGAGA TGG	____*______________*	Wild type
		2: intron:OGDH	TGGTCCTGCTGCTG CTGAGT AGG	___*_______________*	Wild type
		3: intergenic:RP11-426D19.1-MSI2	GGGGCTGGCTGCTG CTGAGG AGG	*____**_____________	Wild type
		intergenic:USP47-DKK3	TTGTGCTGCTGCTG CTGAGG TGG	_*_**_______________	Wild type
SMC3	Total T21 and Dis21 SMC3+/- mutant clones carrying or not the GATA1s and MPLW515K	4: intron: PPAP2A	TGATAAAATTAGAT AAGCCC *TGG*	*________*___**_____	Wild type
		4: intron: SNX29	GGAAAAAAGAAGA CAAGCCC *AGG*	___*____**____*_____	Wild type
		4: exon: ZNF839	GGATAAAATCAGA CGAATCT *AGG*	_________*______**_*	Wild type
		4: exon: LINC00371	GGATACAGAGAGA CGACCCC *GGG*	_____*_**_______*___	Wild type
		3: intron:Y_RNA	AGATAAAATGAAA CCAGCCC AGG	*__________*__*_____	Wild type

		3: intron:MBD5	GGATAAAGTGAGA AGAGACC AGG	−−−−−−−*−−−−−*−−−*−−	Wild type

Table S2: List of culture reagents with manufacturers and catalog numbers (related to methods).

**Product**	**Manufacturer**	**Catalog number**
(+)-JQ-1	CliniSciences, Nanterre, France	HY-13030
1-thioglycerol	Sigma, St Quentin Fallavier, France	#M6145
CellTrace Violet Cell Proliferation Kit	ThermoFisher scientific, Illkirch, France	#C34557
CHiR 99021 trihydrochloride	TOCRIS, Rennes, France	#4953
hBMP4	Peprotech, Neuilly-Sur-Seine, France	#AF-120-05ET
hFGF-basic	Peprotech, Neuilly-Sur-Seine, France	#100-18B
hFLT3L	Celldex Therapeutics Inc, Needham, USA	/
hIL-6	Peprotech, Neuilly-Sur-Seine, France	#200-06
hSCF	Biovitrum AB, Stockholm, Sweden	/
hTPO	Kirin Brewery, Tokyo, Japan	/
hVEGF	Peprotech, Neuilly-Sur-Seine, France	#100-20
Essential 8	Gibco, Villebon sur Yvette, France	#A1517001
Essential 8 Flex	Gibco, Villebon sur Yvette, France	#2858501
L-glutamine	Gibco, Villebon sur Yvette, France	#25030081
N-truncated vitronectin	Gibco, Villebon sur Yvette, France	#A14700
PlasmoTest - Mycoplasma Detection Kit	Invitrogen, Toulouse, France	#rep-pt1
Ruxolitinib	Euromedex, Strasbourg France	#S1378
StemPro-34 SFM	Gibco, Villebon sur Yvette, France	#10639011
StemPro Accutase	Gibco, Villebon sur Yvette, France	#A1110501
Penicillin/Streptomycin	Gibco, Villebon sur Yvette, France	#15140122
Y-27632 dihydrochloride	TOCRIS, Rennes, France	#1254

Table S3: List of CRISPR Cas9 sgRNA and primers for PCR amplification used (related to methods).

**Locus**	**Forward (5' to 3')**	**Reverse (5' to 3')**
**CRISPR/Cas9 sgRNA**		
GATA1	**CACCG**TGCGGCACTGGCCTACTAC	**AAAC**GTAGTAGGCCAGTGCCGCA**C**
SMC3	**CACCG**GGATAAAATGAGACGAGCCC	**AAAC**GGGCTCGTCTCATTTTATCC**C**
MPL	**CACCG**CTGGCCTGCTGCTGCTGAGG	**AAAC**CCTCAGCAGCAGCAGGCCAG**C**
**PCR from genomic DNA - Genotype/Sanger sequencing**		
GATA1	CACACCAGAATCAGGGGTTT	CAGTTGAGGCAGGGTAGAGCCCCGTC
SMC3	TTCTTCCACCTCTCTCCCCA	AGCAAGGACCACATGGCTAA
MPL	**P1_MPL**: TAGGATACGTAGCTCTCTGAGGTG	**P2_MPL**: GTCACAGAGCGAACCAAGAATG
		**P3_Cherry**: GAAGCGCATGAACTCCTTGATG
**PCR from genomic DNA - Off targets**		
GATA1		
ASAP1	TGGTGCTGATTATAGGAGTGAC	AAGCGTTTCCCCTCATCTCAG
DNAH8	GATGTGCCTGTCAGTCACGATC	TTGACTGGAATGTTCTCTCACTC
SREBF2	TGCTCAAGAAAGTCTTCCAGTG	TAAGGAGGAGAGGTAGCATGAGA
XPNPEP2	TTCATTGACCATGCCTTGCCTTG	ACCTTCAACGTGGACTCAGGTC
THOC5	TTCCTTACTTCCGGTTCTCTATG	CAGGCTACATTACCACAATCAAG
SMC3		
PPAP2A	AAGCCAGTGGTTCTTAAACTTGC	ATAGCTCTCCCCATGGACACTG
LINC00371	TACTGGCTCAGGCAACTCTCTC	GATGGCCTATTCTATAAGTCCAC
SNX29	AGAAGTCTTGCCTGAGAATGGAG	AGTGCCCAGAAGGCAGTTATTTC
ZNF839	ACATTGTCACAGTGACTGATGCAG	TTCATCTGTGCAATATTCACATTA
Y_RNA	CAAGATAAGGTTATTATAACTAC	TGTTAGACCTTTGATACCTAAAG
MBD5	TTATCCTAACTCAAGTTAACCAG	CATTCCACTGTATTGTGTCACT
MPL		
BACH2	CTCACGTTAGCGCTGTGCGAC	GTCAACAATGTAGCGATTGAGAG
BRAP	AACAACCAGTGACACACTCATAG	TCCTGCGCGCATGCGTAAACC
GABRG1	AAGACCAACCTCACCCCTCTAC	TCTGCTGGGAGTCGCATCCTAC
TTC34	AATGCAGTCGCCTGGTCCAAAG	ATGGAGCTGGATTCAGAGGAC
AC138647.1-AC104417.1	CATCCACCTCTCCAACAGAGTAA	TTATTCACTCAGCTCCACCTTG
AC106860.1-RP11-234O6.2	AGCATTATCCACAATCTGACTGA	CCACCTGTGTTTACTTAAGGTG
DVL1	TGGCTGTGGACATCCTCACA	AAGGGACAGGGAATAGCCTG
SDK2	AAGTACCATCGTGACCAAGGAT	CTTGGTGTGCTTTGGCAAAGAG
RP11-719N22.2_SRP	GTATCACTGTTGGAGAGAATATC	CAAATGCACTGGTGTATACATAC
TBC1D22A-RP1-111J24.1	ACACGGAATAAAGATCAGTGACA	AAGCAGGTGCTTGACAGATGTG
NSDHL-ZNF185	CAAGAGAGCAATTCCTCACCC	AGAGGCTATGTCTCCTCTCTGA
OGDH	ATCAGATGCTTTAAGTCTGTGG	AAGGCTTAAGTGTTGGCCTTAAC
RP11-426D19.1-MSI2	CCGACTTCTTCCAATAGCTTAG	CTGCATGTTATTAGCAGCCTTC
USP47-DKK3	AATCTAACCTCTCCTCCCTCAA	TGTAGTGTGAGGGACAAAGACA

Table S4: Growth factors and their concentrations used for hematopoietic differentiation from iPSC (related to method).

	**BMP4**	**CHIR**	**VEGF**	**bFGF**	**SCF**	**Flt3L**	**TPO**	**IL6**
Day 0	10ng/mL	930 ng/mL	50ng/mL					
Day 1	10ng/mL		50ng/mL	20ng/mL				
Day 4			15ng/mL	5ng/mL				
Day 6			50ng/mL	50ng/mL	50ng/mL	5ng/mL		
Day 7			50ng/mL	50ng/mL	50ng/mL	5ng/mL	50ng/mL	10ng/mL
Day 8			50ng/mL	50ng/mL	50ng/mL	5ng/mL	50ng/mL	10ng/mL
Day 11			50ng/mL	50ng/mL	50ng/mL	5ng/mL	50ng/mL	10ng/mL
Day 13			50ng/mL	50ng/mL	50ng/mL	5ng/mL	50ng/mL	10ng/mL

Table S5: List of antibodies (related to methods).

Antibody	Manufacturer	Catalog number	Working dilution	Technique
CD34 - PE Cy7	BD Biosciences, Isère, France	#560710 / 581	1/100	Flow cytometry
CD43 - APC	BD Biosciences, Isère, France	#560198 / 1G10	1/100	
CD41a - APC	BD Biosciences, Isère, France	#559777 / HIP8	1/100	
CD42a - PE	BD Biosciences, Isère, France	#558819 / ALMA.16	1/100	
CD42b - BV421	BD Biosciences, Isère, France	#740075 / HIP1	1/100	
CD33 - PE	BD Biosciences, Isère, France	#561816 / WM53	1/100	
CD117_PE Cy7	BD Biosciences, Isère, France	#339217	1/100	
GATA1/GATA1s	Abcam, Amsterdam, Netherlands	#ab133274	1/100	
CD41a	Fisher scientific, Illkirch, France	#BDB555465 / HIP8, BD	1/100	Immunofluorescence
vWF	Dako, Les Ulis, France	#A0082 / P04275	1/1000	
vWF	Abcam, Amsterdam, Netherlands	#ab194405 / 3E2D10	1/200	
CD63	Abcam, Amsterdam, Netherlands	#ab118307	1/100	
Beta1-Tubulin	Abcam, Amsterdam, Netherlands	#ab204947 / 2A1A9	1/100	
GFP	Abcam, Amsterdam, Netherlands	#ab183734 / EPR14104	1/200	
IgG (H+L) anti-Mouse Alexa 488	Thermo Fischer Scientific	#A11029	1/500	
IgG (H+L) anti-Rabbit Alexa 488	Thermo Fischer Scientific	#A11008	1/500	
IgG (H+L) anti-Mouse Alexa 546	Thermo Fischer Scientific	#A11003	1/500	
IgG (H+L) anti-Rabbit Alexa 546	Thermo Fischer Scientific	#A11010	1/500	
GATA1/GATA1s	Abcam, Amsterdam, Netherlands	#ab11852	1/1000	Western blot
SMC3	Abcam, Amsterdam, Netherlands	#ab228782	1/1000	
Histone 3	Abcam, Amsterdam, Netherlands	#ab1791	1/5000	
PCNA	Santa Cruz	#sc-56	1/5000	
GATA1/GATA1s	Abcam, Amsterdam, Netherlands	#ab11852	1/100	CUT & Tag

Table S6: List of the primers used for qRT-PCR (related to methods).

**Locus**	**Forward (5' to 3')**	**Reverse (5' to 3')**
**Taq Man**		
NFE2 probe	CATCACCGAGCTGCAGGGTCTGA	
NFE2	TACAGCTGTCCACTTCAGAGC	GGCTTGGGGCTCAAATGATG
HPRT probe	CTTGCTGGTGAAAAGGACCCCACGA	
HPRT	GGCAGTATAATCCAAAGTTGGTCAA	TCAAATCCAACAAAGTCTGGCTTATA
**SYBR Green**		
CAPN2	TGCTCTTTGTGCATTCAGCC	AGCGCTTCATAGCATCCGTT
TUBB1	CTCAGAGCAAGGATGCGTGA	CAAGTCGATCCCGTGTTCCT
RAB27B	GGGAAGACATTTCTTTATAG	TTCACAATAAGCATTTGCTTGC
TBXAS1	GCGGAGAGACTTCCTCCAAATG	CTGCAATCACCATGTCCAGATAG
GP1BA	TCCTCCATGGGGCTAGAAGA	CCTTACACTCGCCTCACTCA

Full unedited blot for Figure S1B

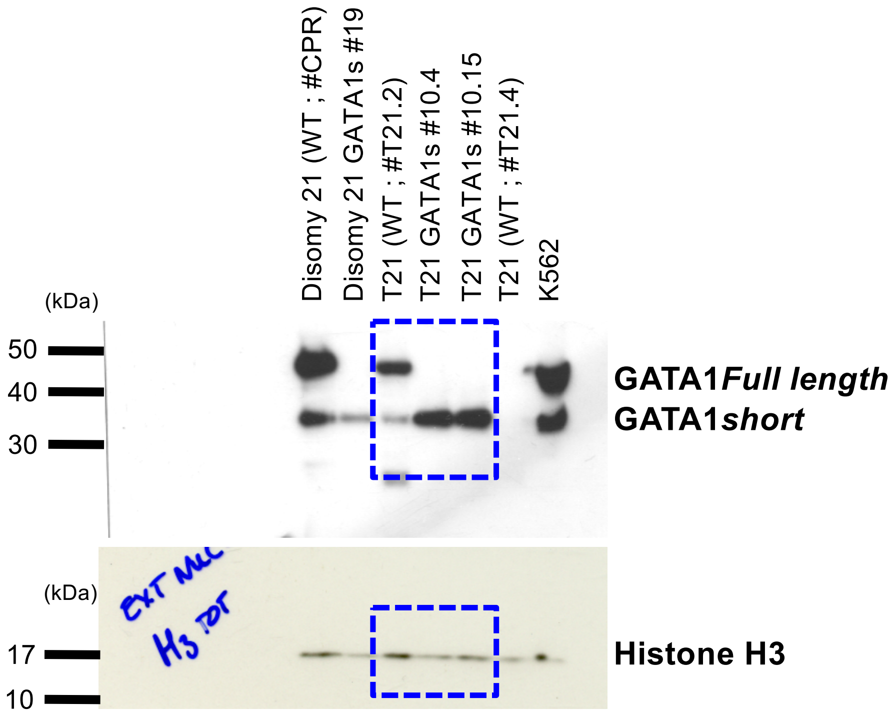

Full unedited gel for Figure S1E

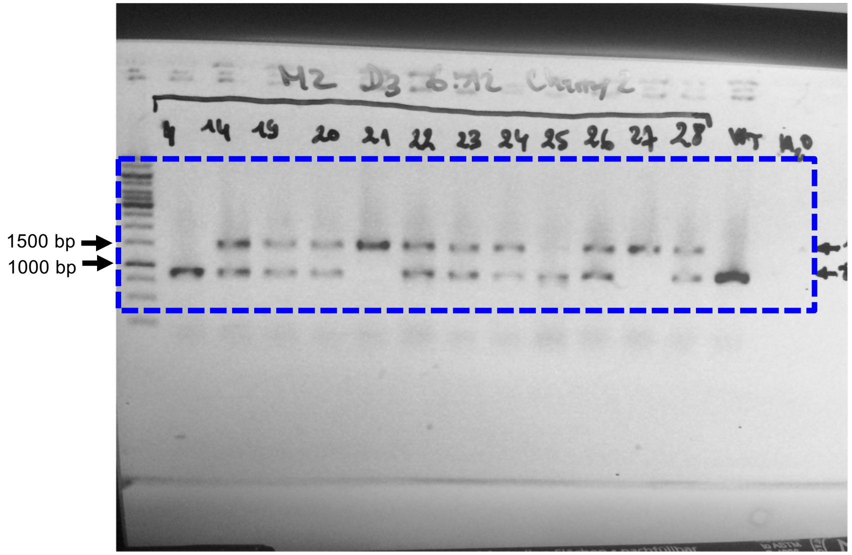

Full unedited gel for Figure S1J

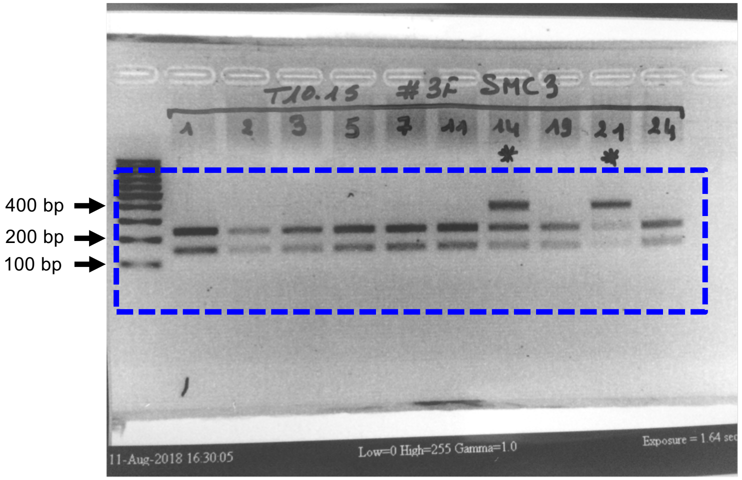

Full unedited blots for Figure S1L

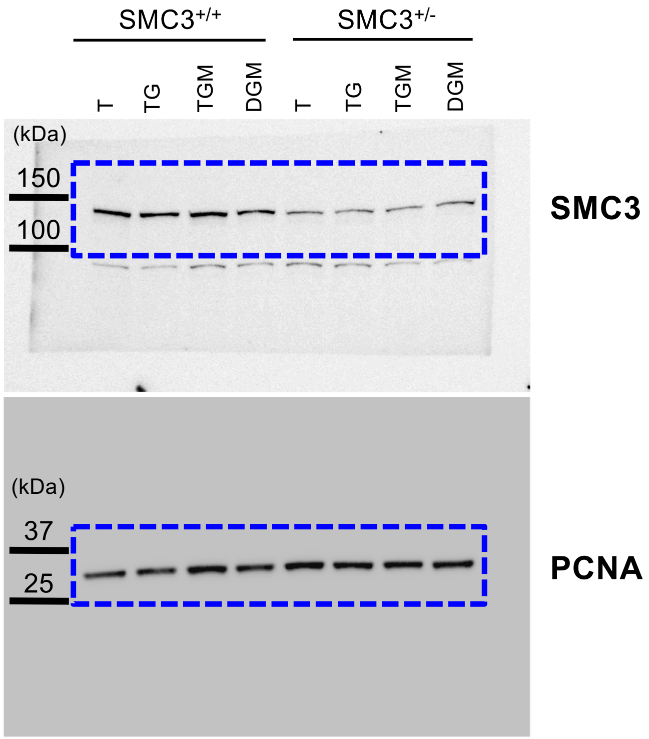

Supplement: Supplemental data [file jci-132-156290-s009.pdf]
